# Efficacy and safety of ephedra-containing oral medications: a systematic review, meta-analysis, and exploratory dose–response analysis for weight reduction

**DOI:** 10.3389/fphar.2024.1397247

**Published:** 2024-10-30

**Authors:** Hyeongyu Cho, Jeewoo Oh, Hongmin Chu, Hanbit Jin, Jungtae Leem

**Affiliations:** ^1^ College of Korean Medicine, Wonkwang University, Iksan, Republic of Korea; ^2^ Mapo Hongik Korean Medicine Clinic, Seoul, Republic of Korea; ^3^ Research Center of Traditional Korean Medicine, College of Korean Medicine, Wonkwang University, Iksan, Republic of Korea

**Keywords:** ephedra, overweight, obesity, dose–response analysis, systematic review and meta-analysis

## Abstract

**Introduction:**

Despite the widespread use of ephedra in various forms, including food supplements and herbal prescriptions, comprehensive studies reviewing its efficacy and safety across different countries are lacking.

**Methods:**

We systematically searched 5 electronic databases and conducted a meta-analysis of 16 randomized controlled trials (RCTs) on ephedra-containing oral medications (EOMs), performing a dose–response analysis for weight loss.

**Results:**

The meta-analysis results revealed a statistically significant reduction in the body mass index (BMI) (MD: 1.5 kg/m2; 95% CI: −2.46 to −0.54) and secondary outcomes like body weight (BW) and waist circumference (WC). The dose–response analysis indicated a correlation between ephedra and weight reduction. The safety analysis showed no significant difference in adverse effects between the treatment and control groups (RR = 0.99, 95% CI = 0.80 ∼ 1.21, and p = 0.90).

**Discussion:**

In conclusion, EOMs demonstrated effectiveness in promoting weight loss, and the dose–response analysis indicated a correlation between ephedra and weight reduction. However, additional research is necessary due to the limited number of studies and inconsistent results among the assessment criteria. Moreover, if prescribed by traditional medicine physicians within the permissible daily ephedrine dosage range of 150 mg set by the Food and Drug Administration (FDA) and monitored by healthcare professionals, the risk of severe adverse events is likely to be minimal.

**Systematic Review Registration:**

https://www.crd.york.ac.uk/prospero/display_record.php?RecordID=387895, identifier CRD42023387895.

## 1 Introduction

In recent years, the number of obese people has increased significantly owing to sedentary lifestyles and high-calorie diets (Manson et al., 2004). The World Health Organization (WHO) defines being overweight as having a body mass index (BMI) of 25 or more and obesity as a BMI of 30 or more (WHO Regional Office for Europe, 2022). Globally, the population of overweight and obese adults has increased by 27.5% between 1980 and 2013 (Ng et al., 2014), representing 30% of the global population in 2015 (Bomberg et al., 2017). Obesity is a metabolic disorder characterized by the abnormal accumulation of excess adipose tissue in the body, extending beyond excess body weight (Engin et al., 2017). It can cause hemodynamic problems and abnormalities in the heart’s structure and function. Individuals with metabolic syndrome, including obesity, have a 40%–60% higher mortality rate from cardiovascular disease (Sundström et al., 2006; Powell-Wiley et al., 2021). Obesity is also a risk factor for increasing the prevalence of other diseases, such as metabolic syndrome, type 2 diabetes, hypertension, coronary artery disease, cancer, and stroke (Hasani-Ranjbar et al., 2009; Spiotta and Luma, 2008). According to a 2006 survey, individuals with obesity spend more than 42% more on healthcare annually than those with a healthy weight (Caroline and Apovian, 2016). Based on current trends, healthcare expenses related to obesity in the United States are expected to reach $48–66 billion annually by 2030 (Wang YC. et al., 2011). In conclusion, obesity has become a serious problem in modern society, reducing life expectancy and adding to social and economic burdens such as increased healthcare costs and decreased productivity (Wang YC. et al., 2011; Blackburn and Walker, 2005; Flegal et al., 2005; Kim et al., 2018).

Pharmacological, surgical, and lifestyle interventions are commonly used to treat obesity (Kissane and Pratt, 2011). Sibutramine, which is frequently prescribed and approved for long-term use, may lead to elevated blood pressure and cause side effects such as insomnia and nausea (Tziomalos et al., 2009). Orlistat causes gastrointestinal upset, and rimonabant is known to increase the incidence of mental conditions, such as depression and anxiety (Rucker et al., 2007). Furthermore, the use of phentermine–topiramate is contraindicated in individuals with cardiovascular disease, thereby limiting its application to specific patient populations (Hasani-Ranjbar et al., 2009; Ioannides-Demos et al., 2006). Surgical therapies are more invasive, require a long recovery period, and may lead to long-term metabolic complications such as osteoporosis, hypoglycemia, and nutrient imbalances (Jammah, 2015). Behavioral modifications, such as regular exercise, dietary adjustments, and low-energy diets, augment energy expenditure compared to physical inactivity or limited calorie consumption (Wadden et al., 2020). Although effective in achieving sustained weight loss while minimizing adverse effects and weight regain, lifestyle interventions are challenging to uphold during and after treatment because of issues with personal adherence (Hasani-Ranjbar et al., 2009; Leibel et al., 1995). The usage of dietary supplements, alongside lifestyle interventions, is steadily increasing. However, there is a significant lack of clinical evidence regarding their effectiveness and safety (Poddar et al., 2011).

Therefore, the demand for East Asian traditional medicines (EATMs) is growing among the general public (Davis et al., 2011; Ojukwu et al., 2015). Herbal medicine is gaining popularity as a treatment option for obesity management in individuals seeking EATMs (Park et al., 2012). Herbal medicine treats obesity through various mechanisms, including augmentation of metabolic rates, carbohydrate metabolism modulation, fat absorption inhibition, appetite suppression, and serotonin modulation (He et al., 2020). Comparing clinical trials of herbal medicine and lifestyle interventions for obesity treatment showed that the combined utilization of herbal medicine and lifestyle interventions demonstrated a more substantial weight loss effect than other interventions (Park et al., 2012).

Among other herbal medicines, ephedra has gained popularity as a treatment for obesity in the United States and other countries, particularly since the 1972 report of its weight-loss effects with caffeine. However, concerns surrounding the adverse effects of ephedra prompted the Food and Drug Administration (FDA) to ban its use in foods in 2004 (Miao et al., 2020; Mehendale et al., 2004). Although adverse events associated with ephedra have decreased following its use after the FDA ban (Zell-Kanter et al., 2015), it is still used in traditional medicine for weight loss and other therapeutic purposes in Asian countries, such as China, Republic of Korea, and Japan, often in combination with other herbs (EBM-based Obesity KMCPG Development Committee, 2016; Uneda et al., 2022; Lee et al., 2020). Ephedra contains alkaloids, flavonoids, and tannins, with the primary bioactive compounds being alkaloids, i.e., 1-ephedrine, 1-methyl-ephedrine, and 1-norephedrine (Miao et al., 2020). Ephedra exerts its effects by stimulating the sympathetic nervous system, inducing energy expenditure, and modifying the gut microbiota in obese individuals (Miao et al., 2020; Alraei, 2010; Kim et al., 2014).

The weight-loss effects and mechanisms of ephedra are well known, leading to their widespread use in clinical practice. Despite the widespread use of ephedra in various forms, including food supplements and herbal prescriptions, comprehensive studies reviewing its efficacy and safety in different countries still need to be performed. Further investigation is necessary to evaluate the effectiveness and safety of ephedra in clinical practice. This study performed a systematic review and meta-analysis to comprehensively evaluate randomized controlled trials (RCTs) of ephedra-containing oral medications (EOMs) for weight loss and obesity treatment, regardless of the formulation or country. This meta-analysis assessed the effectiveness and safety of EOMs in terms of BMI, body weight (BW), and waist circumference (WC). Moreover, considering the insufficient amount of research on the dosage and effects of ephedra, we aimed to explore the dose–response relationship of ephedra using both quantitative and qualitative methodologies.

## 2 Methods

This systematic review and meta-analysis were conducted in accordance with the Preferred Reporting Items for Systematic Reviews and Meta-Analyses (PRISMA) 2020 statement (Mj et al., 2021) and the recommendations of the Cochrane Handbook for Systematic Reviews of Interventions (Higgins et al., 2019). The study protocol was registered in PROSPERO (crd42023387895).

### 2.1 Search strategy

The initial search was conducted on 10 August 2022 using the electronic databases PubMed, Excerpta Medica Database (Embase), Cochrane Central Register of Controlled Trials (CENTRAL), Allied and Complementary Medicine Database (AMED), and Cumulative Index of Nursing and Allied Health Literature (CINAHL). The second search was performed on 15 December 2023. The search terms used were “obesity,” “EATM,” “natural products,” “ephedra,” and “RCT” (Supplementary Table S1). These three categories were combined using the AND Boolean operator with keywords appropriate for each database. In addition, clinical practice guidelines and literature reviews on herbal treatments for obesity were examined. Only articles that met the inclusion criteria were selected for review.

### 2.2 Inclusion and exclusion criteria

#### 2.2.1 Study design

Our study included only RCTs involving human participants. Pilot studies that did not report these results were also excluded. There were no restrictions on the blinding used in the RCTs; however, crossover studies were excluded if the necessary information could not be extracted during data analysis.

#### 2.2.2 Participant characteristics

We included studies of overweight and obese patients with a BMI of 25 or more according to WHO criteria, regardless of sex and age (WHO Regional Office for Europe, 2022). Underlying condition were not restricted; however, individuals who used EOMs for growth or weight gain were excluded from the study.

#### 2.2.3 Types of interventions in the treatment and control groups

The treatment group included all interventions involving ephedra, regardless of whether they were combined with herbal prescriptions, single agents, or food supplements. However, we excluded interventions that contained only a partial component of ephedra, such as ephedrine, and cases where the prescription composition was not presented, making it unclear whether ephedra was included.

We only included study designs that used the same lifestyle interventions, such as exercise and diet, in the treatment and control groups. There were no restrictions on the use of other medications.

#### 2.2.4 Outcome measures

The primary outcome was the BMI, calculated as weight divided by the square of height. Secondary outcome variables were weight (kg) and waist circumference (cm) (Delpino and Figueiredo, 2021). Studies that focused solely on blood test values, including serum leptin concentration, were excluded. The frequency and types of adverse events were also assessed.

### 2.3 Study selection and data extraction

#### 2.3.1 Study selection

Two independent reviewers, HC and JO, initially screened the abstracts and titles to identify potentially eligible articles, which were further evaluated by reviewing the full text. No language restrictions were imposed. Disagreements between the two reviewers (HC and JO) were discussed with a third independent reviewer (JL) to reach a consensus.

#### 2.3.2 Data extraction

HC and JO, summarized the basic information (author, year, and country), blinding, number of participants included (randomized/completed), number of patients by sex, interventions implemented in the treatment and control groups, treatment period, and primary outcome measures. The composition of the intervention, the daily dose, and the pharmaceutical company are described in detail. If necessary data were missing or errors were identified, we contacted the original authors. We also documented the type and number of adverse events, the number of dropouts, and the reasons for dropping out. If any of these details were not explicitly reported in the article, we indicated them as “NR” (not reported).

### 2.4 Assessment of risk of bias

Two reviewers, HC and JO, independently assessed the risk of bias (ROB) according to the RoB 2 tool published by the Cochrane Collaboration (Jac et al., 2019). The tool assesses the risk of bias in five areas: randomization process, deviations from the intended interventions, missing outcome data, measurement of the outcome, and selection of the reported results. Each of the five areas and the risk of bias were rated as low, of some concern, or high. In accordance with the methodology for systematic reviews of interventions (Higgins et al., 2019), if the two reviewers could not reach an agreement on the assessment of ROB, the disagreement was discussed with a third independent reviewer (JL) to reach a consensus.

### 2.5 Data analysis and quantitative synthesis

Statistical analyses were performed using RevMan 5.4 software (Cochrane Training, London, United Kingdom) and R software (version 4.2.1; R Foundation for Statistical Computing, Vienna, Austria).

#### 2.5.1 Assessment of the overall effect size

Each study was evaluated for the total effect size of BMI, BW, and WC changes. A random-effects model accounted for intervention composition, dose, and duration variations between studies. Continuous outcomes of change are presented as mean differences (MDs) with 95% confidence intervals (CIs), and dichotomous outcomes are presented as risk ratios (RRs) with 95% confidence intervals. When standard deviations (SDs) were not reported, they were estimated from confidence intervals, and statistical results were obtained directly from the original authors by acquiring raw data. A random-effects model was also used to assess the number of participants with adverse events and dropouts for the total effect size. Statistical significance was set at *p* < 0.05.

#### 2.5.2 Assessment of heterogeneity and meta-regression analysis

To evaluate the presence of heterogeneity among the included studies, we used the chi-square test and I^2^ statistic. Statistically significant heterogeneity was defined as *p* < 0.10 for the chi-square test. Additionally, an I^2^ value >50% indicated substantial heterogeneity in the study sample (Higgins et al., 2003). Furthermore, we performed meta-regression analyses to investigate potential associations between study-level covariates and the observed statistical heterogeneity (Sg and Jp, 2002). We conducted univariate meta-regression analyses on selected covariates, including daily ephedrine dose, treatment period, baseline BMI, BW, and WC. The analysis used the DerSimonian–Laird methodology (DerSimonian and Laird, 1986; DerSimonian and Laird, 2015), and Wald-type tests were applied for statistical evaluation. The ephedrine dose of ephedra was not reported in the study and was estimated to be 5.25 mg/g, based on the minimum dose specified in the Korean Pharmacopoeia (Jang et al., 2007).

#### 2.5.3 Bubble plot

Two bubble plots were generated to visually depict the relationship between the covariates and changes in the outcome measures. A four-dimensional graph was plotted, in which the *x*-axis represents the outcome at baseline, the *y*-axis represents the daily ephedrine dose, the bubble color denotes the treatment period, and the bubble size corresponds to the magnitude of the outcome reduction. In addition, a three-dimensional graph was plotted to represent the relationship between ephedra intake and efficacy. The *x*-axis in this graph represents the outcome measure at baseline, the *y*-axis represents the total ephedrine dose, and the bubble size represents the reduction in the outcome measure.

#### 2.5.4 Assessment of publication bias

Publication bias was assessed for outcome variables, including the 10 studies in the meta-analysis (Sterne et al., 2011). To determine the potential for publication bias, we presented contour-enhanced funnel plots of the included studies for each outcome (BMI and BW) (Peters et al., 2008). Publication bias was assessed by performing Egger’s test on the observed asymmetry in the funnel plot (Egger et al., 1997).

## 3 Results

**FIGURE 1:**
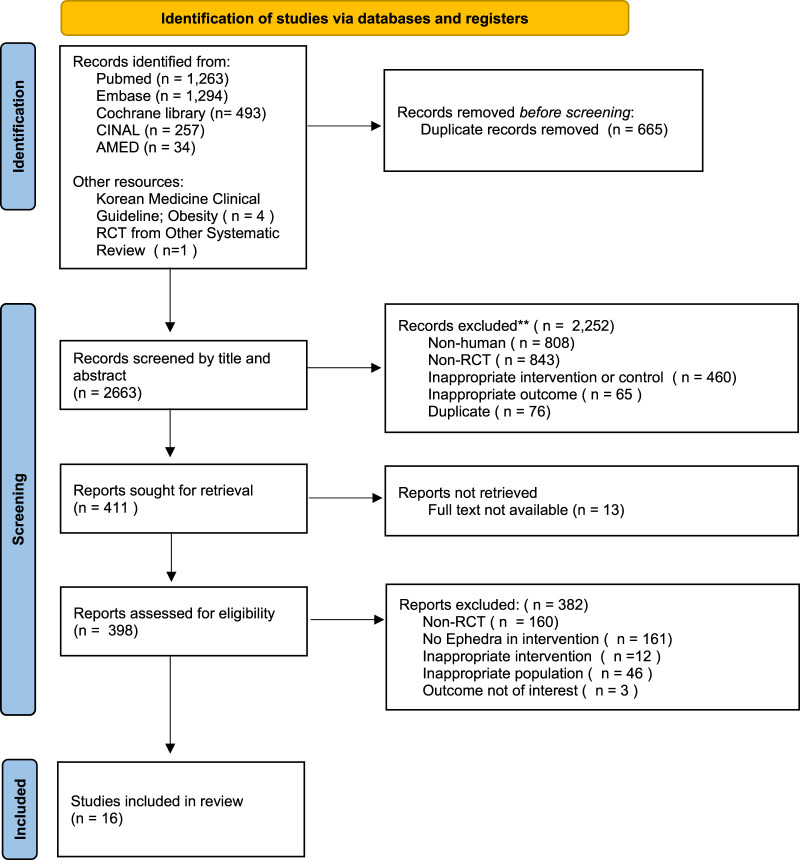
PRISMA flowchart of the study selection.

Following the previous search strategy, we searched five electronic databases and identified 3,149 articles, excluding duplicates and retractions. These were reviewed with articles from other sources, resulting in the selection of 16 articles that met the predetermined inclusion criteria (Figure 1).

### 3.1 General characteristics of the included studies

Seven of the 16 studies analyzed were conducted in the Republic of Korea, 5 in the United States, 2 in Japan, and 2 in China, with 1,460 participants (748 in the treatment group and 712 in the control group). Cheon et al. (2020) and Hioki et al. (2004) studied only women, whereas the others neither restricted sex nor reported sex-based criteria. The mean age of the patients in each study ranged from 30.8 to 60 years.

Regarding the intervention of the treatment group, only Kim et al. (2008) used a ephedra as a single herb as the intervention. At the same time, the remaining studies used a combination of ephedra and other ingredients and herbs in forms such as EATM prescriptions or dietary supplements. In addition, all studies used a placebo as a control, except for Hackman et al. [50], who used a multi-nutrient supplement without ephedra or caffeine as a control. Azushima et al. (2015) used conventional therapy for hypertension in both groups, whereas Cheon et al. (2020), Hackman et al. (2006), Coffey et al. (2004), and Feng-Hao et al. (2012) did not use or mention combination therapy. The remaining studies used the usual care for obesity in both the treatment and control groups. The group that received 5% of the treatment group’s dosage was considered the placebo group.

The treatment period varied from 4 to 24 weeks, and the main outcome measures included BMI, BW, and WC, which were also the selection criteria for this study. Additionally, various metabolic and drug toxicity markers were assessed, including serum lipids, blood glucose, aspartate transaminase (AST), alanine transaminase (ALT), and blood urea nitrogen (BUN). The daily dose of ephedra varied from 0.6 to 14 g. The details of each study are summarized in Table 1, and the composition and dosage of the interventions are summarized in Supplementary Table S2.

**TABLE 1:** Summary of RCTs.

Author, year, location	Blinding	Subject N (randomized/completed)	Sex (M/F)	Age mean (SD)	Type of interventions	Treatment	Control	Treatment period	Ephedra herb Daily dose (g)/total dose (g)	Main outcome measure^a^
Cheon, 2020, Republic of Korea	A, P	T) 76/62 C) 73/60	T) 0/76 C) 0/73	T) 42.5 (10.7) C) 43.7 (76)	Herbal medicine (prescription)	Euiiyin-tang (薏苡仁汤)	Placebo	12 weeks	3.99/335.16	BW, BMI, WC, HC, WHR, TC, LDL, HDL, triglycerides, TFA, VFA, SFA, VFA/SFA, CRP, KQQOL, KEAT-26, SRRS, BP, PR, AST, ALT, BUN, creatinine, daily intake of calories, SRI
Azushima, 2015, Japan	O	T) 54/42 C) 52/46	T) 28/26 C) 29/23	T) 59.2 (14.5) C) 60.0 (12.9)	Herbal medicine (prescription)	FFTSS (防风通圣散) + conventional therapy	Placebo + conventional therapy	24 weeks	4.6%/NR	Ambulatory BP and HR, body weight, BMI, abdominal circumference, glucose–lipid metabolism, renal function, adipokines, oxidative stress
Park, 2014, Republic of Korea	A, P	T) 55/42 C) 56/40	NR	T) 41.56 (8.62) C) 39.21 (10.12)	Herbal medicine (prescription)	FFTSS (防风通圣散) + UC	Placebo + UC	8 weeks	0.6/33.6	BW, BMI, WC, BFP, BFM, RMR, fasting BST, TC, HDL, TG, KOQOL, SBP, DBP, PR, ALT, AST, BUN, creatinine
Jian, 2014, China	NR	T) 30/25 C) 30/27	NR	NR	Herbal medicine (prescription)	Pelian Mahuang + UC	Placebo + UC	12 weeks	14/1,176	BW, BMI, WHR, TG, TC, LDL
Park, 2013, Republic of Korea	A, P	T) 58/41 C) 55/45	T) 7/50 C) 10/45	T) 39.2 (9.5) C) 38.8 (10.1)	Herbal medicine (prescription)	Taiyin Tiaowei Decoction (太阴调胃汤) + UC	Placebo + UC	12 weeks	3.75/315	BW, BMI, WC, HC, WHR, cholesterol, body fat compression, C-reactive protein, BP, PR, AST, ALT, BUN, creatinine
XU, 2012, China	A, P	T) 70/67 C) 50/45	T) 19/48 C) 11/34	T) 60 (1) C) 60 (1)	Herbal medicine (prescription)	FFTSS (防风通圣散) (7.5 g extract)	FFTSS (防风通圣散) (7.5 g, 5% of active BTS-added substitute)	8 weeks	2.4/134.4	BW, BFP, BP, HR
Park, 2011, Republic of Korea	A, P	T) 55/42 C) 56/40	21/145	T) 41.56 (8.62) C) 39.21 (10.12)	Herbal medicine (prescription)	FFTSS (防风通圣散) (Hanpoong Pharm. Ltd.) + UC	Placebo + UC	8 weeks	1.2/67.2	BW, BMI, WC, BFP, BFM, BP, PR, TG, TC, HDL, fasting BST, RMR
Li, 2010, Republic of Korea (a)	A, P	T) 28/23 C) 24/18	T) 6/29 C) 4/28	T) 42.2 (8.1) C) 40.0 (9.4)	Herbal medicine (prescription)	Hanpoong Taeumjowitang ext. granule + UC	Placebo + UC	12 weeks	3.75/315	BW, BMI, WC, WHR, TG, TC, LDL, HDL, TFA, VFA, SFA, VSR, KOQOL, KEAT-26, AST, ALT, BUN, creatinine
Li, 2010, Republic of Korea (b)	A, P	T) 18/15 C) 18/16	T) 4/14 C) 3/15	T) 39.11 (10.66) C) 39.44 (10.57)	Herbal medicine (prescription)	FFTSS (防风通圣散) (Hanpoong Pharm. Ltd.) + UC	Placebo + UC	4 weeks	1.2/33.6	BW, WC, WHR, BMI, BFM, BFP, FFM, BMR, TFA, VVFA, SFA, VSR, TC, TG, HDL, LDL, glucose, CRP, leptin, adiponectin, KOQOL, SRI, KEAT-26, AST, ALT, γ-GT, BUN, creatinine
Kim, 2008, Republic of Korea	A, P	T) 41/21 C) 39/16	NR	T) 33.8 (7.9) C) 30.8 (7.4)	Herbal medicine (single agent)	*Ephedra sinica* + UC	Placebo + UC	8 weeks	12/672	RMR, BMI, WHR, BFP, FFM, AST, ALT, blood urea nitrogen, creatinine, T-chol, TG
Hackman, 2006, United States	A, P	T) 29/19 C) 32/23	T) 5/47 C) 9/41	T) 38.4 (1.1) C) 35.5 (0.9)	Health supplement	Multinutrient supplement (containing ephedra, caffeine, etc.)	Multinutrient supplement (without ephedra and caffeine)	36 weeks	0.5/126 (ephedrine)	BW, BMI, Body fat, BP, HR, ECGs, TC, HDL, LDL, TG, fasting glucose, fasting insulin, HOMA-IR, leptin, adiponectin, ghrelin
Coffey, 2004, United States	A, P	T) 52/44 C) 50/42	T) 5/47 C) 9/41	T) 44.9 (9.1) C) 42.1 (10.9)	Health supplement	Active product	Placebo	12 weeks	0.75/63	BW, BFP, BFM, BMI, WC, TC, TG, BP, PR
Greenway, 2004, United States	A, P	T) 20/12 C) 20/19	T) 4/16 C) 3/17	T) 46.8 (2.8) C) 45.3 (1.9)	Health supplement	Dietary supplement + UC	Placebo + UC	12 weeks	0.9 (8%)/75.6	BW, PR, BP, TG, HDL, TG, DXA, lean tissue
Hioki, 2004, Japan	A, P	T) 44/41 C) 41/40	T) 0/41 C) 0/40	T) 52.6 (14.0) C) 54.8 (12.5)	Herbal medicine (prescription)	FFTSS (防风通圣散) + UC	Placebo + UC	24 weeks	0.24/4.032 (ephedrine)	BW, BFM, abdominal visceral fat, abdominal subcutaneous fat, BP, HR, WC, HC, TG, T-chol, LDL, HDL, uric acid, HbA1c, fasting glucose, OGTT 2 h glucose, glucose AUC 120, fasting insulin, OGTT 2 h insulin, insulin AUC 120, HOMA-IR
Boozer, 2002, United States	A, P	T) 83/46 C) 84/41	T) Female 78% C) Female 86%	T) 44.5 (12.4 years) C) 46.0 (12.2)	Health supplement	Herbal ephedra/caffeine + UC	Placebo + UC	6 months	0.15/2.520 (ephedrine)	BW, BFM, WC, HC, BP, HR, Holter monitor data, TG, LDL, HDL, T-chol, glucose
Boozer, 2001, United States	A, P	T) 35/24 C) 32/24	T) 6/29 C) 4/28	T) 42.2 (8.1) C) 40.0 (9.4)	Health supplement	Active preparation + UC	Placebo + UC	8 weeks	0.72/4.032 (ephedrine)	BW, BFM, WC, HC, BP, HR, cholesterol, HDL, LDL, TG, glucose

^a^ Main outcome measure.

^b^ If only the ephedrine dose is listed and not ephedra dose, then the ephedrine dose is listed.

A, assessor-blind; BFM, body fat mass; BFP, body fat percent; BMI, body mass index; BMR, basic metabolic rate; BW, body weight; C, control group; FFM, fat-free mass; FFTSS, Fangfeng Tong Sheng San; HDL, high-density lipoprotein; HR, heart rate; KEAT-26, Korean Eating Attitude Test-26; KOQOL, Korean version of the obesity-related QOL scale; LDL, low-density lipoprotein; NR, not reported; O, open-label; P, participant blind; PR, pulse rate; RMR, resting metabolic rate; SFA, subcutaneous fat area; SRI, stress response inventory; T, treatment group; TFA, total fat area; TC, total cholesterol; TG, triglyceride; UC, usual care; VFA, visceral fat area; VSR, VFA/SFA ratio; WC, waist circumference; WHR, waist-to-hip ratio.

### 3.2 Results of the risk of bias assessment

**FIGURE 2:**
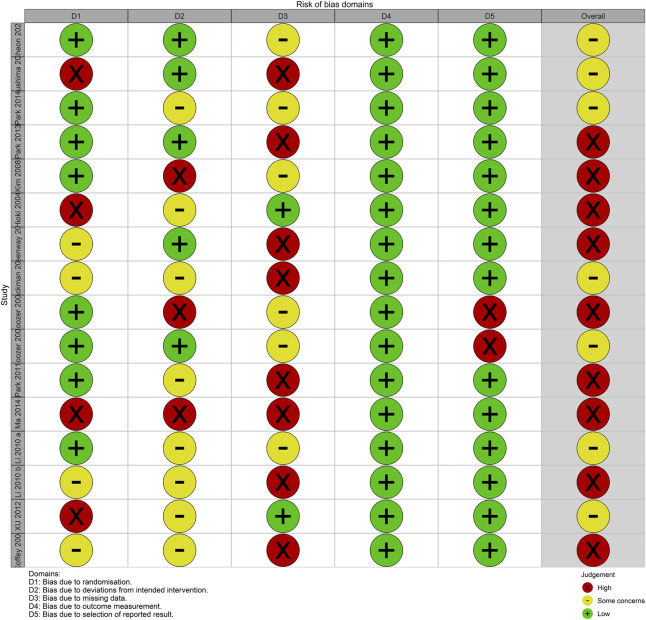
Risk of bias summary for the randomized controlled trials included in this study.

None of the analyzed studies demonstrated a low overall risk of bias, whereas all studies had some concerns or a high overall risk of bias. The risk of bias for the outcome measurement was low because the outcome measure in all studies was an objective metric. Except for two studies (Boozer et al., 2001; Boozer et al., 2002) that reported measurements of height and weight without reporting BMI as an outcome, all other studies reported all outcomes described in the *Methods*. Therefore, the risk of bias in the selection of reported results was mostly low. However, in some studies (C et al., 2004; Azushima et al., 2015; Feng-Hao et al., 2012; MA et al., 2014), a third party did not perform the randomization process or was not properly blinded, raising concerns about the potential for bias in the randomization process. In addition, the risk of bias was high for deviations from the intended interventions and missing outcome data owing to the occurrence of adverse effects that could be inferred from the intervention, and a high number of dropouts for unclear reasons (Figure 2).

### 3.3 Meta-analysis of intervention effects

**FIGURE 3:**
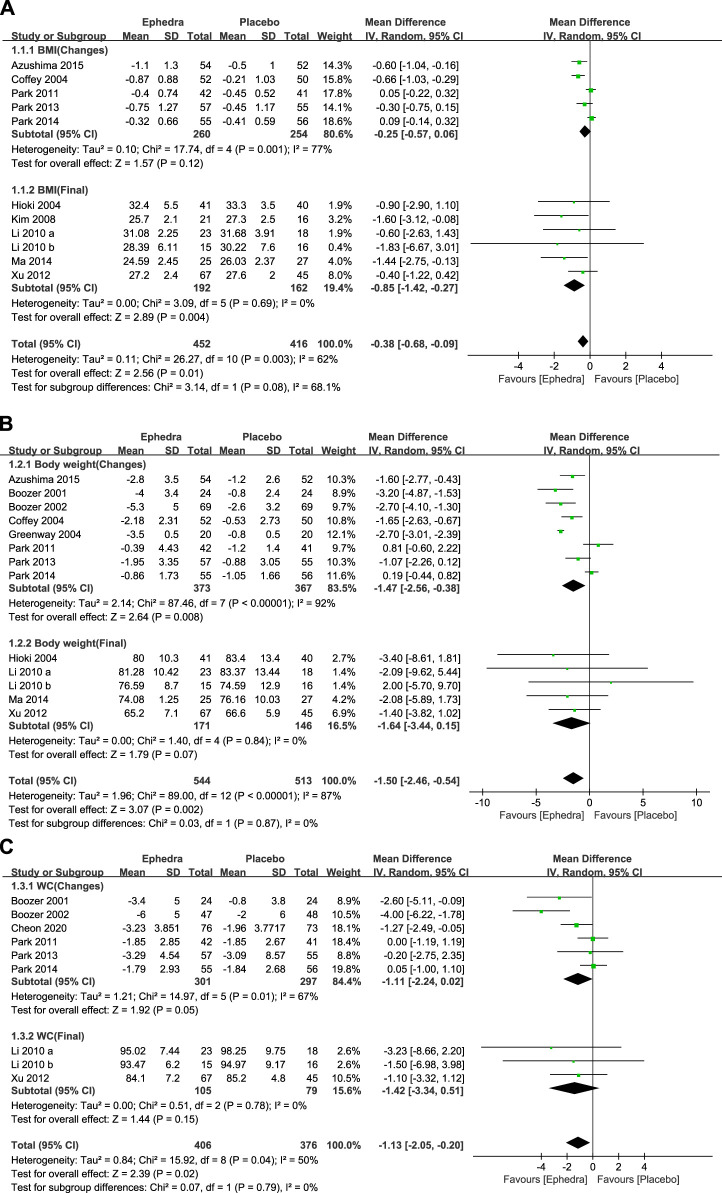
**(A)** Forest plot showing the effect of ephedra on BMI; **(B)** forest plot showing the effect of ephedra on BW; and **(C)** Forest plot showing the effect of ephedra on WC.

A meta-analysis was conducted to examine the effectiveness of EOMs in reducing BMI, BW, and WC. First, 11 studies reported the change in the BMI as an outcome measure, of which 5 reported the amount of change and SD value, while 6 reported the final value and SD value. Forest plots were generated to present each subgroup’s changes and final values, and the overall effects were pooled. The study by Hackman et al. (2006) was excluded because the SD values and raw data were not available. The meta-analysis revealed that EOMs resulted in a statistically significant additional reduction in the BMI compared to the control group (MD = −0.38 kg/m^2^; 95% CI= −0.68 to −0.09) (Figure 3A).

In addition, 13 studies reported changes in BW as an outcome measure, of which 8 reported changes and SD values, while 5 reported final values and SD values. The study by Hackman et al. (2006) was excluded for the same reason. The meta-analysis showed that EOMs resulted in a statistically significant additional reduction in BW compared to the control group (MD = −1.5 kg; 95% CI= −2.46 to −0.54) (Figure 3B).

Nine studies reported changes in WC as an outcome measure, of which six reported change and SD values and three reported final and SD values. The meta-analysis presented that EOMs resulted in a statistically significant additional reduction in WC compared to the control group (MD = −1.13 cm; 95% CI= −2.05 to −0.20) (Figure 3C).

### 3.4 Adverse events reported

The studies by Kim et al. (2008), Coffey et al. (2004), Boozer et al. (2002), and Feng-Hao et al. (2012), which did not report the number of adverse events in the treatment groups, were excluded, the remaining 12 studies were examined. Adverse events were classified into several categories: cardiac, gastrointestinal, serum hepatic enzyme levels, headache/neuropsychiatric, autonomic hyperactivity, gynecological, and dermatological symptoms. The types and numbers of adverse events are summarized in Table 2. The number of dropouts due to these adverse effects was also included. The most frequently reported adverse events were headache and neuropsychiatric symptoms, with headache being the most common. Additionally, the number of adverse events, participants who experienced adverse events, dropouts, and reasons for dropouts in both the treatment and control groups for all 16 studies are summarized in Table 3.

**TABLE 2:** Summary of adverse events in the treatment group.

Classification	Symptom	Number of symptoms	Number of dropouts caused by adverse events	Sum of occurrences
Heart symptoms	Heartburn/chest pain	8	1	8
Gastrointestinal symptoms	Constipation	2	—	52
	Diarrhea	20	2	
	Gastrointestinal	2	—	
	Dyspepsia and epigastric pain	14	1	
	Gastric irritation	1	—	
	Nausea and vomiting	13	1	
Serum hepatic enzyme level	Elevation of serum hepatic enzyme level	1	—	1
Headache/neuropsychiatric symptoms	Headache	30	—	85
	Dull head	—^a^	—	
	Insomnia	20	3	
	Dizziness	8	1	
	Nervousness	19	1	
	Irritability	5	3	
	Poor concentration	2	—	
	Neuropsychiatric	1	—	
Autonomic hyperactivity	Palpitations	26	8	73
	Decreased appetite	22	—	
	Dry mouth	25	—	
Gynecological symptoms	Delayed menstrual period	—	—	—
Dermatological	Herpes zoster	1	—	4
	Allergic dermatitis	1	—	
	Hair loss	1	—	
	Skin problems	1	—	
	Urticaria and multiple premature ventricular contractions	—	3	
Oral symptoms	Oral	1	—	29
	Difficulty concentrating	—	—	
	Other type of pain	1	—	
	Blurred vision	1	—	
	Fatigue	7	—	
	Energy increased	19	—	
	Cold	—	—	
	Tonsillitis	—	—	
	Otitis media	—	—	
	Soreness	—	1	
	Urticaria	—	—	
	Cholelithiasis	1	—	

These data are derived from the PRISMA flowchart or from the dropout information listed in each paper. This shows the number of participants who experienced adverse events and dropped out.

^a^ The symptoms of side effects are known, but the number of occurrences is not.

**TABLE 3:** Summary of adverse effects and dropouts.

Study_ID	AEs in treatment group N = (total AE occurrence/number of participants who experienced AEs)	AEs in placebo group N = (AE n/AE participant n)	Treatment group dropouts N = dropout n	Placebo group dropouts N = dropout n	Number of subjects (randomized/completed)
Cheon, 2020, Republic of Korea	N = 5/8 Headache 1 (moderate) Diarrhea 1 (mild) Herpes zoster 1 (mild) Cholelithiasis 1 (mild) Allergic dermatitis 1 (mild) Aspartate aminotransferase increased 0 Alanine aminotransferase increased 0 Concussion 0 Peripheral swelling 0 Hypertonic bladder 0 Uterine leiomyoma 0 Uterine polyp 0	N=8 / 6 Headache 0 Diarrhea 1 (mild) Herpes zoster 0 Cholelithiasis 0 Allergic dermatitis 0 Aspartate aminotransferase increased 1 (mild) Alanine aminotransferase increased 1 (mild) Concussion 1 (severe) Peripheral swelling 1 (mild) Hypertonic bladder 1 (mild) Uterine leiomyoma 1 (mild) Uterine polyp 1 (moderate)	N=14 never showed up 5 withdrew consent 8 adverse event 1	N=13 never showed up 7 withdrew consent 3 adverse event 3	T) 76/62 C) 73/60
Azushima, 2015, Japan	N = 3/NR 3 minor adverse events (gastric irritation, constipation, and elevation of the serum hepatic enzyme level)	N= 0/NR	N=12 lost to follow-up 8 withdrew consent 1 adverse event 2 became pregnant 1	N=6 lost to follow-up 4 withdrew consent 2	T) 54/42 C) 52/46
Park, 2014, Republic of Korea	N=15/NR Dyspepsia, epigastric pain 7 Headache 2 Diarrhea 3 Nausea, vomiting 2 Insomnia 0 Palpitations 1	N=4/NR Dyspepsia, epigastric pain 3 Headache 1 Diarrhea 0 Nausea, vomiting 0 Insomnia 0 Palpitations 1	N=13 personal choice 7 protocol violation 7 epigastric pain 1 dyspepsia 1	N=16 personal choice 4 protocol violation 7 palpitations 1 dyspepsia 1	T) 55/42 C) 56/40
Park, 2013, Republic of Korea	N= 0/0	N= 0/0	N=16 lost to follow-up 7 protocol violation 3 subject withdrawal 6	N=10 lost to follow-up 5 protocol violation 1 subject withdrawal 3 concurrent disease 1	T) 58/41 C) 55/45
Kim, 2008, Republic of Korea	N= NR /NR Palpitations 0 -> 1 Headache 7 -> 7 Dull head 8 -> 4 Tremor 0 -> 0 Insomnia 0 -> 4 Dizziness 4- > 4 Nervousness 2 -> 1 Nausea 0 -> 2 Vomiting 0 -> 2 Anorexia 0 -> 1 Constipation 8 -> 12 Dysuria 0 -> 0 skin rash 0 -> 0 Dry mouth 0 -> 6 Breathlessness 0 -> 0	N= NR /NR Palpitations 0 -> 0 Headache 2 -> 0 Dull head 3-> 0 Tremor 1 -> 0 Insomnia 1 -> 2 Dizziness 4 -> 0 Nervousness 2 -> 1 Nausea 0 -> 1 Vomiting 0 -> 0 Anorexia 0 -> 0 Constipation 4 -> 3 Dysuria 1- > 0 Eruption 0 -> 1 Dry mouth 1 -> 1 Breathlessness 0 -> 0	N=20 moved out 1 other disease 4 personal choice 15	N=23 moved out + travel 4 other disease 4 pregnancy 1 nausea 1 personal choice 13	T) 41/21 C) 39/16
Hioki, 2004, Japan	N=0/0	N=0/0	N=3 non-compliance because of diarrhea (BF, which contains Natrium Sulphuricum and Rhei Rhizoma, promotes bowel movement)	N=1 non-compliance 1	T) 44/41 C) 41/40
Greenway, 2004, USA	N=20/NR Respiratory 11 Pain 1 Gastrointestinal 2 Oral 1 Genitourinary 0 Headache 2 Nerve compression 0 Hair loss 1 Skin problems 1 Neuropsychiatric 1 Arrhythmia 0	N=27/NR Respiratory 8 Pain 3 Gastrointestinal 5 Oral 4 Genitourinary 3 Headache 1 Nerve compression 1 Hair loss 0 Skin problems 0 Neuropsychiatric 1 Arrhythmia 1	N=8 follow-up 5 withdrew consent 2 breast tenderness 1	N=1 scheduling conflict 1	T) 20/12 C) 20/19
Coffey, 2004, USA	N= NR/78 NR	N=NR/56 PTARE: Exacerbated depression Atrial fibrillation Exacerbation of asthma	N=8 Low back pain 1 Compression fracture of L1 1 Unable to meet protocol criteria 1 Withdrawn for a protocol violation or noncompliance 1 Withdrew consent 2 Lost to follow up 2	N=8 Emesis 1 Elevated blood pressure 1 Hypothyroidism 1 Withdrew consent 3 Lost to follow up 2	T) 52/ 44 C) 50/42
Hackman, 2006, USA	N=123/NR Decreased appetite 22 Dizziness 5 Dry mouth 14 Increased energy 19 Fatigue 7 Headache 16 Insomnia 7 Nausea 7 Nervousness 13 Palpitations 13	N=35/NR Decreased appetite 1 Dizziness 3 Dry mouth 4 Increased energy 3 Fatigue 4 Headache 13 Insomnia 2 Nausea 3 Nervousness 1 Palpitations 1	N=10 Non-compliance 1 Insomnia 1 Nervousness 1 Lost to follow up 1 dizziness 1 headaches 2 medication 2 surgery 1	N=8 Lost to follow up 4 Personal conflict 3 Medication 1	T) 29/19 C) 32/23
Boozer, 2002, USA	N=NR/NR constipation diarrhea difficulty concentrating dizziness dry mouth heartburn insomnia anxiety upset stomach	N=NR/NR constipation diarrhea difficulty concentrating dizziness dry mouth heartburn insomnia anxiety upset stomach	N=37 protocol 3 non-compliance 4 personal choice 14 Chest pain 0 Loud heartbeat 1 Palpitations 3 Elevated blood pressure 2 Irregular heartbeat 1 Multifocal ventricular event 1 Ventricular event 1 Ventricular runs of five or more fast heartbeats 1 Anxiety 1 Disorientation 0 Dizziness 0 Insomnia 2 Irritability 2 Bad taste 1 Dry mouth 1 Gastroesophageal reflux disorder 1 Nausea 1 Gallbladder removal 0 Elevated creatinine 1	N=43 protocol 4 non-compliance 3 personal choice 24 Chest pain 2 Loud heartbeat 0 Palpitations 2 Elevated blood pressure 3 Irregular heartbeat 1 Multifocal ventricular event 1 Ventricular event 1 Ventricular runs of five or more fast heartbeats 1 Anxiety 0 Disorientation 1 Dizziness 1 Insomnia 0 Irritability 0 Bad taste 1 Dry mouth 0 Gastroesophageal reflux disorder 0 Nausea 0 Gallbladder removal 1 Elevated creatinine 0	T) 83/46 C) 84/41
Boozer, 2001, USA	N=59/NR Irritability 5 Dizziness 3 Insomnia 13 Anxiety 6 Headache 7 Blurred vision 1 Poor concentration 2 palpitation 1 Constipation 1 Diarrhea 2 Upset stomach 0 Heartburn 4 Nausea 2 Dry mouth 11 Chest pain 1	N=43/NR Irritability 3 Dizziness 1 Insomnia 9 Anxiety 6 Headache 4 Blurred vision 2 Poor concentration 3 palpitations 1 Constipation 4 Diarrhea 1 Upset stomach 2 Heartburn 2 Nausea 0 Dry mouth 4 Chest pain 1	N= 11 Palpitations 4 Palpitations + chest pain 1 Elevated BP 2 Irritability 1 Personal choice 3	N= 8 Personal choice 6 Recurring medical condition 2	T) 35/24 C) 32/24
Park, 2011, Republic of Korea	N=15/NR Dyspepsia, epigastric pain 7 Headache 2 Diarrhea 3 Nausea, vomiting 2 Palpitation 1	N=4/NR Dyspepsia, Epigastric pain 3 Diarrhea 1	N=13 NR	N=16 NR	T) 55/42 C) 56/40
MA Jian, 2014, China	N=1/NR mild diarrhea	N=0/NR	N=5 withdrawn for a protocol violation or noncompliance 4	N=3 withdrawn for a protocol violation or noncompliance	T) 30/25 C) 30/27
Ji-Eun Li, 2010, Republic of Korea	N=NR/5 5 people in the treatment group with 9 symptoms : cold, tonsillitis, Otitis media, muscle pain, fatigue, delayed menstrual period, insomnia, urticaria, multiple ventricular premature contractions	N=NR/3 3 people in the placebo group with 5 symptoms: acute sore throat, otalgia, laceration of the finger, endometrial polyp, diarrhea	N=5 never showed up 2 personal choice 1 lost to follow-up 1 adverse effect 1	N=6 never showed up 2 unable to meet protocol criteria 1 personal choice 1 lost to follow-up 1 other disease 1	T) 28/23 C) 24/18
Ji-Eun Li, 2010, Republic of Korea	sore, chest pain 3 more than three episodes of diarrhea per day without stomach ache 10	NR	N=3 NR	N=2 NR	T) 18/15 C) 18/16
Xu, 2012, China	NR	NR	N=3 fever, liver function change	N=5 constipation, fever, refusal	T) 70/67 C) 50/45

**FIGURE 4:**
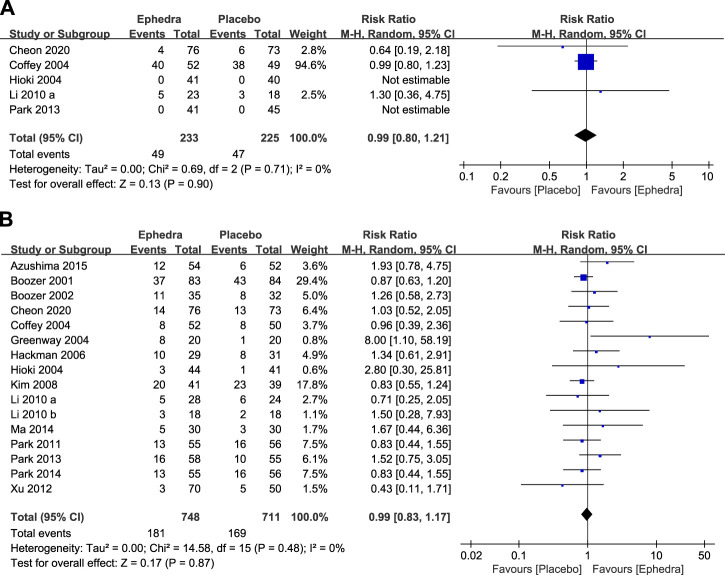
**(A)** Forest plot for participants who experienced adverse effects and **(B)** Forest plot for dropouts.

A meta-analysis was performed using data from five studies that reported the number of participants who experienced adverse events. There was no statistically significant difference between the treatment and control groups (RR = 0.99; 95% CI = 0.80–1.21) (Figure 4A). A meta-analysis of the number of dropouts across all selected studies showed no statistically significant differences between the treatment and control groups (RR = 0.99; 95% CI = 0.83–1.17) (Figure 4B).

### 3.5 Meta-regression and bubble plot results

In the meta-analysis of post-treatment changes in the BMI, BW, and WC, the chi^2^ test yielded a value of *p* < 0.10, and the I^2^ values were 62, 87, and 50%, respectively, indicating high heterogeneity among the studies. Therefore, a univariate meta-regression analysis explored the relationship between heterogeneity and covariates. For change in BMI, both the daily dose of ephedrine (QM = 18.5143, *p* < 0.001, tau^2^ = 0, I^2^ = 0%, and R^2^ = 100%) and duration (QM = 4.4590, *p* = 0.0347, tau^2^ = 0.0313, I^2^ = 29.34%, and R^2^ = 69.50%) explained the heterogeneity but not the initial BMI (QM = 1.0719, *p* = 0.3005, tau^2^ = 0.0625, I^2^ = 44.22%, and R^2^ = 39.20%). For changes in BW, the daily dose of ephedrine (QM = 14.0831, *p* = 0.0002, tau^2^ = 0.3087, I^2^ = 37.74%, and R^2^ = 85.93%) explained the heterogeneity, but the period (QM = 2.3635, *p* = 0.1242, tau^2^ = 1.5880, I^2^ = 83.14%, and R^2^ = 27.62%) and initial BW (QM = 3.4327, *p* = 0.0639, tau^2^ = 1.4732, I^2^ = 81.47%, and R^2^ = 32.85%) did not explain the heterogeneity. Finally, for changes in WC, the period (QM = 9.8250, *p* = 0.0017, tau^2^ = 0, I^2^ = 0%, and R^2^ = 100%) explained heterogeneity as a variable, but the daily dose of ephedrine (QM = 2.7709, *p* = 0.0960, tau^2^ = 0.4313, I^2^ = 33.55%, and R^2^ = 48.71%) and initial WC (QM = 0.0012, *p* = 0.9724, tau^2^ = 1.1167, I^2^ = 55.66%, and R^2^ = 0%) did not explain the heterogeneity (Table 4). The results of the meta-regression analysis are visualized using bubble plots (Supplementary Figure S1).

**TABLE 4:** Meta-regression analysis (univariate).

Covariate	Coefficient	SE	Z-value	*p-*value	95% CI
BMI					
Daily ephedrine dose*	−15.0705	3.5025	−4.3028	<.0001	−21.9353 to −8.2058
Treatment period*	−0.0953	0.0451	−2.116	0.0347	−0.1837 to −0.0068
Initial BMI	−0.0541	0.0523	−1.0353	0.3005	−0.1566 to 0.0483
BW					
Daily ephedrine dose*	−34.9725	9.3192	−3.7527	0.0002	−53.2378 to −16.7072
Treatment period	−0.1415	0.0920	−1.5374	0.1242	−0.3219 to 0.0389
Initial BW	−0.1065	0.0575	−1.8528	0.0639	−0.2192 to 0.0062
WC					
Daily ephedrine dose	−38.0242	22.8427	−1.6646	0.0960	−82.7952 to 6.7467
Treatment period*	−0.2211	0.0705	−3.1345	0.0017	−0.3594 to −0.0829
Initial WC	−0.0038	0.1098	−0.0346	0.9724	−0.2189 to 0.2113

* Covariate demonstrated a statistically significant association with observed heterogeneity (*p-*value < 0.05).

Unit used for analysis: body mass index (kg/m^2^); body weight (kg); daily ephedrine dose (g); treatment period (weeks); waist circumference (cm).

BMI, body mass index; BW, body weight; CI, confidence interval; SE, standard error; WC, waist circumference.

**FIGURE 5:**
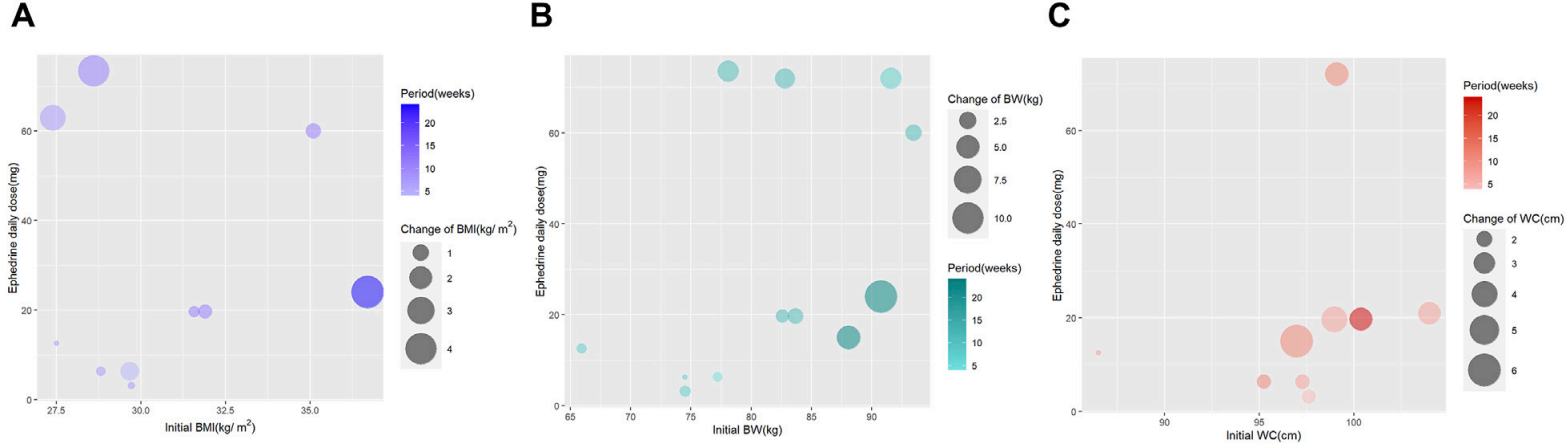
**(A)** Bubble plot for body mass index change; **(B)** bubble plot for body weight change; and **(C)** bubble plot for waist circumference change.

We also generated bubble plots for 10 studies reporting changes in BMI, 12 studies reporting changes in BW, and 9 studies reporting changes in WC, all of which reported the daily doses of ephedra or ephedrine. We developed a four-dimensional graph with the *x*-axis indicating the baseline measurement of the outcome variable, the *y*-axis indicating the daily dose of ephedrine, the color of the bubble indicating the study period, and the size of the bubble representing the reduction in the outcome measure (Figures 5A–C). In the same study, we developed another type of bubble plot for each outcome measure. The *x*-axis represents the outcome measure at baseline, the *y*-axis represents the total ephedrine dose, and the bubble size represents a reduction in the outcome measure (Supplementary Figure S2).

### 3.6 Assessment of publication bias results

**FIGURE 6:**
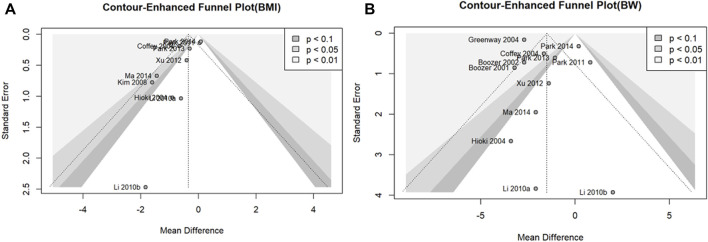
**(A)** Contour-enhanced funnel plot for BMI. **(B)** Contour-enhanced funnel plot for BW.

We assessed the potential for publication bias in the outcome variables, BMI and BW, using 10 or more studies included in the meta-analysis. Contour-enhanced funnel plots showed asymmetry in all outcome measures, confirming the possibility of publication bias (Figures 6A, B). Egger’s test was used to assess publication bias. There was no statistically significant publication bias for BW (*p* = 0.3361); however, there was a possibility of publication bias for BMI (*p* = 0.0300).

## 4 Discussion

### 4.1 Summary of findings

This study aimed to determine the efficacy and safety of EOMs in overweight and obese individuals. The 16 RCTs that included ephedra in interventions for individuals with a BMI of 25 or greater were identified, and their characteristics, interventions, and adverse effects were summarized. A meta-analysis was performed on the outcome measures of BMI, BW, and WC to determine the statistical significance of these effects. The results showed a statistically significant reduction in all outcome measures in the treatment group compared with the control group. A meta-analysis was also performed on adverse events and dropouts and showed no statistically significant difference between the treatment and control groups. Furthermore, a meta-regression analysis was conducted to explore the relationship between heterogeneity in the meta-analysis of effects and covariates. The results indicated that the daily dose of ephedrine and treatment period explained the heterogeneity in BMI change, a daily dose of ephedrine explained the heterogeneity in BW change, and the treatment period explained the heterogeneity in WC change. Additionally, bubble plots were used to visually demonstrate that the covariates were proportionally associated with reductions in the outcome measures (BMI, BW, and WC).

#### 4.1.1 Debate: the efficacy of ephedra on obesity

With the growing popularity of EATM, herbal medicine has received considerable attention as a treatment for obesity. Ephedra is one of the preferred herbal medications for the treatment of obesity, as identified in the Korean Medicine Clinical Practice Guideline for Obesity, which lists several herbal formulas, including Fangfeng Tong Sheng San, Taeumjowitang, Euiiyin-tang, and Chegamuiiyin-tang (Wooltorton and Sibbald, 2002). These formulas have been extensively studied in the Republic of Korea and reported to have clinical effects (Hwang et al., 2007). Previous clinical trials using ephedra have shown that they effectively reduce obesity-related markers such as BW and WC (Boozer et al., 2002). In addition, several systematic reviews have reported statistically significant weight-loss effects in the majority of interventions containing ephedra, further supporting its efficacy as a medication for obesity treatment (Hasani-Ranjbar et al., 2009; Maunder et al., 2020). The results of this systematic review, which synthesized existing studies, support ephedra’s previously reported weight-loss effects. Furthermore, the daily dose of ephedra and the treatment period may have a dose–response relationship, which requires further investigation in additional studies. Moreover, there was a weak association with baseline body weight, suggesting that ephedra may be used for individuals with a BMI of 25 or higher, irrespective of their initial weight status.

The best-known mechanism for the weight-loss effects of ephedra is through the modulation of the sympathetic nervous system to increase metabolism and exercise capacity. In addition, Park et al. (Park SJ. et al., 2022; Park WY. et al., 2022) showed that ephedra plays a dual role in energy metabolism, inhibiting lipogenesis and promoting thermogenesis through browning in the mature state. It has also been reported to exhibit anti-obesity effects by affecting the gut microbiota associated with fat accumulation (Kim et al., 2014). As such, ephedra is a promising drug for treating obesity as it may act not only through sympathetic nervous system stimulation but also other various mechanisms. Research based on the network pharmacology of EOMs is increasingly being conducted. Based on these studies, it is predicted that new mechanisms for the treatment of obesity will be further elucidated that have not been previously predicted and that involve ephedra and other traditional herbal prescriptions, (Jang et al., 2021).

#### 4.1.2 Debate: the safety of ephedra

Ephedra is an herbal medicine that has been the subject of ongoing safety concerns (Bent et al., 2003). Despite these concerns, prior to the 2004 FDA ban, ephedra remained unregulated as a food product in the United States, leading to its widespread misuse. Continued reports of adverse effects eventually prompted the FDA to prohibit the sale of dietary supplements containing ephedra (Palamar, 2011). Similarly, the European Union (EU), Canada, and Australia imposed bans on ephedra-containing food products for the same reasons. Furthermore, the European Food Safety Authority (EFSA) concluded that the variability in ephedrine alkaloid content and distribution across *Ephedra* species makes it difficult to establish a safe daily intake as a food (EFSA Panel on Food Additives and Nutrient Sources added to Food ANS, 2013).

Ephedra is prohibited by global sports organizations such as the International Olympic Committee (IOC) and the National Collegiate Athletic Association (NCAA) due to its performance-enhancing effects and associated health risks (Powers, 2001; Keisler and Hosey, 2005). Additionally, the World Anti-Doping Agency (WADA) includes ephedrine, the active compound in ephedra, on its list of banned substances (WADA, 2023).

In contrast, ephedra continues to be used in East Asian countries for medicinal purposes, where it is listed in the pharmacopeias of each nation and regulated as a pharmaceutical product under specific standards. Its use is controlled through prescriptions by medical professionals (Tang et al., 2023; WADA, 2023; The Minister of Health and Labour and Welfare, 2016; Kim and Oh, 2020). In the Republic of Korea, the Ministry of Food and Drug Safety (MFDS) has prohibited the distribution of ephedra as a food product and has strictly regulated it as a pharmaceutical product (Kim et al., 2006). While this does not guarantee safety from all potential risks, some reports suggest that using ephedra under medical supervision results in fewer side effects than self-administration of dietary supplements containing ephedra (Lee et al., 2020; Mehendale et al., 2004; Lin et al., 2012).

Before the FDA ban on ephedra, there were reports that dietary supplements containing ephedra caused serious adverse events, including death (Haller and Benowitz, 2000), which led to an FDA ban on the sale of such supplements in 2004 (Miao et al., 2020; Mehendale et al., 2004). Approximately half of the adverse effects of ephedra reported to the FDA are cardiovascular, including coronary artery constriction, vasospasm, arrhythmias, and diseases secondary to hypertension due to the overactivation of the sympathetic nervous system (Wooltorton and Sibbald, 2002). Naik and Freudenberger (2004) clinically demonstrated that coronary artery constriction and vasospasm through sympathomimetic effects have the potential to cause myocarditis. This safety concern has resulted in restrictions on its use based on patient characteristics such as heart disease, hypertension, diabetes, anxiety, and glaucoma (Hsing et al., 2006).

These adverse effects are thought to be due to the toxicity of the alkaloids present in ephedra (Tang et al., 2023). Odaguchi et al. (2019) found that alkaloid-free ephedra extracts may contribute to a lower incidence of adverse effects, suggesting that attention should be paid to alkaloids for their safe use. In addition, endogenous catecholamines released by ephedrine directly or indirectly stimulate the sympathetic nervous system, resulting in central nervous system symptoms, such as mental excitement, insomnia, and wakefulness (Maglione et al., 2005). However, the non-alkaloidal components of ephedra have been used to treat obesity, asthma, and pain through antioxidant, anti-inflammatory, and immunosuppressive mechanisms, suggesting their potential for use in various diseases (Powell-Wiley et al., 2021). Nevertheless, the lack of clarity regarding safety issues may account for the recent reduced use of ephedra.

In this study, we reviewed adverse events in RCTs and found no evidence that the treatment group had a significantly higher dropout rate or adverse events. Although this suggests that its use in a controlled environment, such as a clinical trial, does not result in a higher rate of adverse events, further research is required to assess its safety in the real world. However, when prescribed within the FDA-approved daily ephedrine dose range of 150 mg (Kim et al., 2007), as in this study, the risk of severe adverse events from ephedra is considered to be relatively low, as is the case of herbal medicines with medical monitoring.

Among the studies included in this review, Hackman et al. (2006) reported the highest adverse effects, including decreased appetite and increased energy. However, these are common mechanisms for treating obesity (Christoffersen et al., 2022; Shim et al., 2017), and Miao et al. (2020) and Alraei (2010) reported an increase in energy metabolism as a major mechanism for weight loss. Therefore, depending on how adverse effects are defined, it may be possible to overestimate ephedra’s adverse effects. Further consensus among clinicians and researchers is required to determine which symptoms should be considered adverse effects.

#### 4.1.3 Debate: existing studies on EATM dosage and the dose–response relationship of ephedra in the treatment of obesity

Statistical approaches have been used to determine the optimal dose that maximizes the efficacy of EATM treatments. For instance, various studies have explored dose–response relationships in acupuncture. Qin et al. (2019) explored the dose–response relationship between the number of acupuncture treatments and their effectiveness in chronic prostatitis and pelvic pain syndrome, while Xu et al. (2022) used meta-regression to explore the dose–response relationship in major depressive disorders. Similar attempts have been made for EATM. For example, Tai et al. (2022) utilized real-world data from patients with heart failure to demonstrate the dose–response relationship of the Fuzi (Radix Aconiti Lateralis Preparata) dose with the occurrence of composite cardiovascular events and the association of timing with prognosis.

However, no studies have explored the dose–response relationship between herbal medicines and acupuncture in obesity. This need is particularly pronounced in the case of ephedra, given the significant concerns regarding side effects. Therefore, it is important to determine the optimal dose of ephedra that can safely produce therapeutic effects. Our study found an association between the duration or daily dose of ephedra and treatment outcomes; however, owing to the small number of studies and inconsistent results between endpoints, further research is needed.

### 4.2 Strengths and limitations

This study has several strengths. Although previous reviews have examined herbal prescriptions for obesity, as well as single agents like ephedra and ephedrine (Park et al., 2012; Shekelle et al., 2003; Sui et al., 2012), no review has focused solely on ephedra. Hence, this systematic review is the first to comprehensively examine the use of ephedra, including single agents, mixed herbal formulas, and food supplements, for weight loss and treating obesity. Moreover, the selection of studies from diverse countries, formulations, and treatment periods allowed us to extensively evaluate their characteristics, therapeutic effects, and adverse effects. Notably, we summarized the types and frequencies of adverse events reported in the included studies to help identify possible trends in adverse events.

Additionally, dosage, duration, and patient baseline are important considerations in clinical practice. This study used meta-regression and bubble plots to explore the relationship between these factors and treatment effects. The results of the meta-regression analysis showed that the daily dose of ephedrine and the treatment period explained a significant amount of heterogeneity in the therapeutic effect. The bubble plot also illustrates the potential relationship between these factors and their therapeutic effects. These findings may provide a basis for further research into the dose–response relationship between treatment period and ephedra dose in treating obesity.

However, this study should be interpreted with caution due to the following limitations. First, the majority of the interventions included in the studies encompassed ephedra and other herbs or chemicals that may have contributed to the treatment effect. Therefore, it cannot be concluded that the results of this meta-analysis are solely attributable to ephedra. In order to evaluate ephedra in combination formulations, further pharmacokinetic and pharmacodynamic studies are required, considering drug interactions between herbal ingredients (Luo et al., 2020). Currently, various studies, including network pharmacology analyses and pharmacokinetic experiments, are being conducted (Miao et al., 2020; Song et al., 2015; Wei et al., 2014). It is deemed necessary to carry out additional research on the synergistic effects of ephedra in order to establish safe guidelines for its clinical use. However, it is noteworthy that EOMs have been reviewed and found to be effective. In clinical studies of EATM, it is impractical to administer ephedra as the sole agent. Therefore, to obtain data on human subjects, an alternative approach would be to analyze studies on EOMs.

Among the various active compounds in ephedra, ephedrine is recognized for its significant role in weight reduction (Miao et al., 2020). To explore the dose effect, this study assumed that ephedra formulations contained the same amount of ephedrine, 5.25 mg/g, the minimum content threshold suggested by the Korean Pharmacopoeia. However, ephedra formulations comprise natural, unprocessed products, and their ephedrine content varies depending on the origin and time of harvest (Matsumoto et al., 2015). There are also variations in the chemical composition of different species of ephedra, and little is known about the clinical differences caused by different alkaloids (Ibragic and Sofić, 2015). Therefore, the results of this study cannot be directly extrapolated to clinical practice. Given the variability due to individual differences between species, along with the effects and interactions when combined with other herbs and prescriptions, further research is necessary, as previously mentioned.

During the study selection process, several limitations were identified in the selection of outcome measures. First, we focused solely on whether the outcome measures included BMI, body weight, and waist circumference without imposing additional criteria. Consequently, the studies we included did not uniformly address the same liver function tests and metabolic markers, such as AST, ALT, BUN, leptin, and adiponectin, with some studies omitting these as outcome measures. As a result, we were unable to comprehensively evaluate the effects of ephedra on metabolic markers and liver function. Second, BMI, which considers only weight and height, is a measure that does not account for other factors, such as muscle mass. Specifically, in older populations, BMI is not an accurate predictor of obesity as it may underestimate obesity due to decreased muscle mass and increased body fat with age (Batsis et al., 2016).

Meta-regression analyses are deemed more meaningful when the number of studies is large, and it is recommended that at least 10 studies should be included (Higgins et al., 2019). However, this study was an exploratory attempt with few studies, especially for WC, and only nine studies met the recommended criteria. Therefore, only univariate meta-regression analyses were performed, and there were differences in significant covariates depending on the outcome measure. Furthermore, Egger’s test for publication bias recommends 10 or more studies in which the WC does not meet the criteria. In addition, bubble plots are solely visual graphs and do not offer conclusive evidence suggesting causality. Owing to the limitations mentioned above (variable ephedrine content of ephedra, insufficient number of studies, and limitations of bubble plots), we limited our meta-regression and bubble plots to exploratory attempts at determining dose–response relationships of ephedra. Nevertheless, as mentioned in the strengths section, these preliminary attempts provide a foundation for further research.

Finally, although we selected the daily dose of ephedrine as a key covariate, this does not mean that we considered the weight-loss effects of ephedra solely dependent on ephedrine. As mentioned earlier, ephedra is a multicomponent herb, and many different mechanisms explain its effects, with non-alkaloidal components contributing to its effects. However, several selected studies reported only the ephedrine dose rather than the ephedra dose. Therefore, we chose ephedrine as the baseline metric to compare the ephedra doses across studies.

### 4.3 Implications for further research and clinical practice

Adverse effects may be expected to increase with higher doses of ephedra; however, this analysis was limited by the small sample size, which needed to be increased for statistical power. As more high-quality RCTs that include pharmacokinetic studies are conducted, a dose–response analysis of ephedra’s efficacy and adverse effects may suggest a safe and appropriate dose of ephedra using methods such as the restricted cubic spline method (Cj et al., 2022).

However, due to unclear herbal constituents, many RCTs were excluded during the selection process. Even among the selected studies, some did not specify the dose of ephedra. High-quality RCTs that clearly describe the study methods, including constituent herbs and the dose of ephedra, are required to draw useful statistical conclusions in clinical practice. Additionally, there needs to be more uniformity in the presentation of drug ingredients and doses, such as ephedrine or ephedra doses. Thus, an international consensus on labeling standards for intervention composition and dose is needed for RCTs on natural products and herbal medicines.

However, the same drug may elicit different responses. For example, the optimal dosage of warfarin may be influenced by CYP2C9 and VKORC1 genotypes, and leveraging this genetic information in prescribing may mitigate adverse effects (Wang L. et al., 2011). Furthermore, recent studies have attempted to use genetics to predict drug responses, such as detecting drug resistance using gene chips (Yin et al., 2020). In addition, inhibiting and modulating certain genes at the fetal and neonatal stages can lead to a phenotype susceptible to cardiac ischemia (Zhang et al., 2021; Patterson et al., 2010; Lawrence et al., 2011). Integrating these genomic studies with the sensitivity to the effects and adverse reactions of ephedra could eventually lead to the determination of its indications and contraindications through genomic testing.

Exploring the impact of ephedra on different population groups would be valuable as this could offer clinicians a more comprehensive understanding of its use. For example, the effects and pharmacokinetic properties of a drug may vary based on factors such as sex and age (Schwartz, 2003; Davis et al., 2012; Busetto et al., 2009). However, in this study, no restrictions were placed on the population to allow for a more comprehensive analysis. Furthermore, given that our study is a secondary analysis (systematic review) based on a prospective RCT, it does not allow for the detailed assessment of effects across different population groups as we cannot acquire individual patient data. The studies we included aimed to balance the baseline characteristics between treatment and control groups through randomization. Furthermore, our review of existing studies revealed that many had conflicting results or involved small sample sizes, reducing their reliability (Haller and Benowitz, 2000; Samenuk et al., 2002; Gurley et al., 1998). However, as more RCTs incorporate diverse population groups in their designs, or as retrospective studies based on individual patient medical records on ephedra increase, it is anticipated that these avenues will provide broader insights into the effects and use of ephedra.

Metabolic markers such as leptin and adiponectin could serve as alternative indicators of the effect of ephedra on weight reduction or the treatment of metabolic disorders (Klempel and Varady, 2011; Yoon et al., 2011). Conversely, to evaluate safety, several cases have reported that ephedra has the potential to elevate liver enzymes and influence kidney function due to its diuretic effects (Saeed et al., 2019). Therefore, to accurately determine the weight-loss efficacy and safety of ephedra, it is essential to establish guidelines specifying which markers should be included as outcome measures. Incorporating these markers into study designs will contribute to higher-quality research in the future.

In conclusion, this study suggests that EOMs may effectively treat overweight and obesity with higher daily doses or longer treatment periods, possibly resulting in greater efficacy. However, because this may increase the risk of adverse events, clinicians must weigh the tradeoff between effectiveness and adverse effects before prescribing these drugs. We anticipate that further research will contribute to the development of professional and standardized guidelines, ensuring the safe use of ephedra in clinical practice. The necessity to mandate the precise specification of the amounts of ephedra and ephedrine used in clinical research should be thoroughly discussed.

## Data Availability

The original contributions presented in the study are included in the article/Supplementary Material; further inquiries can be directed to the corresponding authors.

## References

[B1] Alraei R. G. (2010). Herbal and dietary supplements for weight loss. Top. Clin. Nutr. 25 (2), 136–150. 10.1097/tin.0b013e3181dbb85e

[B2] Azushima K. Tamura K. Haku S. Wakui H. Kanaoka T. Ohsawa M. (2015). Effects of the oriental herbal medicine Bofu-tsusho-san in obesity hypertension: a multicenter, randomized, parallel-group controlled trial (ATH-D-14-01021.R2). Atherosclerosis 240 (1), 297–304. 10.1016/j.atherosclerosis.2015.01.025 25818388

[B3] Batsis J. A. Mackenzie T. A. Bartels S. J. Sahakyan K. R. Somers V. K. Lopez-Jimenez F. (2016). Diagnostic accuracy of body mass index to identify obesity in older adults: NHANES 1999-2004. Int. J. Obes. (Lond) 40 (5), 761–767. 10.1038/ijo.2015.243 26620887 PMC4854777

[B4] Bent S. Tiedt T N. Odden M C. Shlipak M G. (2003). The relative safety of ephedra compared with other herbal products. Ann. Intern. Med. 138, 468–471. 10.7326/0003-4819-138-6-200303180-00010 12639079

[B5] Blackburn G. L. Walker W. A. (2005). Science-based solutions to obesity: what are the roles of academia, government, industry, and health care? Am. J. Clin. Nutr. 82 (1 Suppl. l), 207S–210S. 10.1093/ajcn/82.1.207S 16002821

[B6] Bomberg E. Birch L. Endenburg N. German A. J. Neilson J. Seligman H. (2017). The financial costs, behaviour and psychology of obesity: a one health analysis. J. Comp. Pathology 156 (4), 310–325. 10.1016/j.jcpa.2017.03.007 28460796

[B7] Boozer C. Daly P. Homel P. Solomon J. Blanchard D. Nasser J. (2002). Herbal ephedra/caffeine for weight loss: a 6-month randomized safety and efficacy trial. Int. J. Obes. 26 (5), 593–604. 10.1038/sj.ijo.0802023 12032741

[B8] Boozer C. Nasser J. Heymsfield S. Wang V. Chen G. Solomon J. (2001). An herbal supplement containing Ma Huang-Guarana for weight loss: a randomized, double-blind trial. Int. J. Obes. 25 (3), 316–324. 10.1038/sj.ijo.0801539 11319627

[B9] Busetto L. Mazza M. Salvalaio S. De Stefano F. Marangon M. Calò E. (2009). Obesity treatment in elderly outpatients: predictors of efficacy and drop-out. Eat. Weight Disord. 14 (2–3), e56–e65. 10.1007/BF03327801 19934638

[B10] Caroline M. Apovian M. D. (2016). Obesity: definition, comorbidities, causes, and burden. Suppl. Featur. Publ. 22 (7 Suppl. l). 27356115

[B11] C H. K Y. T Y. (2004). Efficacy of bofu-tsusho-san, an oriental herbal medicine, in obese Japanese women with impaired glucose tolerance. Clin. Exp. Pharmacol. and physiology 31 (9), 614–619. 10.1111/j.1440-1681.2004.04056.x 15479169

[B12] Cheon C. Song Y. K. Ko S. G. (2020). Efficacy and safety of Euiiyin-tang in Korean women with obesity: a randomized, double-blind, placebo-controlled, multicenter trial. Complementary Ther. Med. 51, 102423. 10.1016/j.ctim.2020.102423 32507436

[B13] Christoffersen B. Ø. Sanchez-Delgado G. John L. M. Ryan D. H. Raun K. Ravussin E. (2022). Beyond appetite regulation: targeting energy expenditure, fat oxidation, and lean mass preservation for sustainable weight loss. Obes. (Silver Spring) 30 (4), 841–857. 10.1002/oby.23374 PMC9310705 35333444

[B14] Cj T. M E. S. Yh Y. Yh T. D C. J H. (2022). The effectiveness of Fuzi in combination with routine heart failure treatment on chronic heart failure patients. J. Ethnopharmacol. 10.1016/j.jep.2022.115040 35121051

[B15] Coffey C. S. Steiner D. Baker B. A. Allison D. B. (2004). A randomized double-blind placebo-controlled clinical trial of a product containing ephedrine, caffeine, and other ingredients from herbal sources for treatment of overweight and obesity in the absence of lifestyle treatment. Int. J. Obes. 28 (11), 1411–1419. 10.1038/sj.ijo.0802784 15356670

[B16] Davis C. Fattore L. Kaplan A. S. Carter J. C. Levitan R. D. Kennedy J. L. (2012). The suppression of appetite and food consumption by methylphenidate: the moderating effects of gender and weight status in healthy adults. Int. J. Neuropsychopharmacol. 15 (2), 181–187. 10.1017/S1461145711001039 21733284

[B17] Davis M. A. West A. N. Weeks W. B. Sirovich B. E. (2011). Health behaviors and utilization among users of complementary and alternative medicine for treatment versus health promotion. Health Serv. Res. 46 (5), 1402–1416. 10.1111/j.1475-6773.2011.01270.x 21554272 PMC3207184

[B18] Delpino F. M. Figueiredo L. M. (2021). Melatonin supplementation and anthropometric indicators of obesity: a systematic review and meta-analysis. Nutrition 91–92, 111399. 10.1016/j.nut.2021.111399 34626955

[B19] DerSimonian R. Laird N. (1986). Meta-analysis in clinical trials. Control Clin. Trials 7 (3), 177–188. 10.1016/0197-2456(86)90046-2 3802833

[B20] DerSimonian R. Laird N. (2015). Meta-analysis in clinical trials revisited. Contemp. Clin. Trials 45 (Pt A), 139–145. 10.1016/j.cct.2015.09.002 26343745 PMC4639420

[B21] EBM-based Obesity KMCPG Development Committee (2016). Korean traditional medicinal clinical practice guidelines of obesity.

[B22] EFSA Panel on Food Additives and Nutrient Sources added to Food (ANS) (2013). Scientific Opinion on safety evaluation of Ephedra species for use in food. Sci. Opin. Saf. Eval. Ephedra species use food 11 (11). 10.2903/j.efsa.2013.3467

[B23] Egger M. Davey Smith G. Schneider M. Minder C. (1997). Bias in meta-analysis detected by a simple, graphical test. BMJ 315 (7109), 629–634. 10.1136/bmj.315.7109.629 9310563 PMC2127453

[B24] Engin A. (2017). “The definition and prevalence of obesity and metabolic syndrome,” in Obesity and lipotoxicity. Editors Engin, A. B. Engin A. (Cham: Springer International Publishing), 1–17.

[B25] Feng-Hao X. Kazuo U. Hiroko O. Masayuki M. Hideki O. (2012). Personalized effects of a Kampo herbal formulation on metabolism-A randomized, double-blind, placebo controlled study of Bohu-tsusei-san-. 東方医学 28 (1), 37–59.

[B26] Flegal K. M. Graubard B. I. Williamson D. F. Gail M. H. (2005). Excess deaths associated with underweight, overweight, and obesity. JAMA 293 (15), 1861–1867. 10.1001/jama.293.15.1861 15840860

[B27] Gurley B. J. Gardner S. F. White L. M. Wang P. L. (1998). Ephedrine pharmacokinetics after the ingestion of nutritional supplements containing Ephedra sinica (ma huang). Ther. Drug Monit. 20 (4), 439–445. 10.1097/00007691-199808000-00015 9712471

[B28] G X. H L. L H. Q X. B H. Z Z. (2022). The dose-effect association between acupuncture sessions and its effects on major depressive disorder: a meta-regression of randomized controlled trials. J. Affect. Disord. 310, 318–327. 10.1016/j.jad.2022.04.155 35504399

[B29] Hackman R. M. Havel P. J. Schwartz H. J. Rutledge J. C. Watnik M. R. Noceti E. M. (2006). Multinutrient supplement containing ephedra and caffeine causes weight loss and improves metabolic risk factors in obese women: a randomized controlled trial. Int. J. Obes. 30 (10), 1545–1556. 10.1038/sj.ijo.0803283 16552410

[B30] Haller C. A. Benowitz N. L. (2000). Adverse cardiovascular and central nervous system events associated with dietary supplements containing ephedra alkaloids. N. Engl. J. Med. 343 (25), 1833–1838. 10.1056/NEJM200012213432502 11117974

[B31] Hasani-Ranjbar S. Nayebi N. Larijani B. Abdollahi M. (2009). A systematic review of the efficacy and safety of herbal medicines used in the treatment of obesity. World J. Gastroenterology WJG 15 (25), 3073–3085. 10.3748/wjg.15.3073 PMC2705729 19575486

[B32] He Z. Barrett L. A. Rizvi R. Tang X. Payrovnaziri S. N. Zhang R. (2020). Assessing the use and perception of dietary supplements among obese patients with national health and nutrition examination survey. AMIA Jt. Summits Transl. Sci. Proc. 2020, 231–240. 32477642 PMC7233063

[B33] Higgins J. P. T. Thomas J. Chandler J. Cumpston M. Li T. Page M. J. (2019). Cochrane Handbook for systematic reviews of interventions. 2nd Edition (Chichester (UK): John Wiley and Sons).

[B34] Higgins J. P. T. Thompson S. G. Deeks J. J. Altman D. G. (2003). Measuring inconsistency in meta-analyses. BMJ 327 (7414), 557–560. 10.1136/bmj.327.7414.557 12958120 PMC192859

[B35] Hsing L. Lee T. Son D. Yeo J. Yang C. Seo E. (2006). Adverse effects of ma-huang according to dose: a randomized double-blind placebo-controlled pilot study. J. Korean Orient. Intern. Med. 27 (1), 188–196.

[B36] Hwang M.-J. Shin H.-D. Song M.-Y. (2007). Literature review of herbal medicines on treatment of obesity since 2000;Mainly about ephedra herba. J. Soc. Korean Med. Obes. Res. 7 (1), 39–54.

[B37] Ibragic S. Sofić E. (2015). Chemical composition of various Ephedra species. Bosn. J. Basic Med. Sci. 15 (3), 21–27. 10.17305/bjbms.2015.539 PMC4594322 26295290

[B38] Ioannides-Demos L. L. Proietto J. Tonkin A. M. McNeil J. J. (2006). Safety of drug therapies used for weight loss and treatment of obesity. Drug-Safety 29 (4), 277–302. 10.2165/00002018-200629040-00001 16569079

[B39] Jac S. J S. Mj P. Rg E. Ns B. I B. (2019). RoB 2: a revised tool for assessing risk of bias in randomised trials. BMJ Clin. Res. 366, l4898. 10.1136/bmj.l4898 31462531

[B40] Jammah A. A. (2015). Endocrine and metabolic complications after bariatric surgery. Saudi J. Gastroenterology Official J. Saudi Gastroenterology Assoc. 21 (5), 269–277. 10.4103/1319-3767.164183 PMC4632250 26458852

[B41] Jang D. Jeong H. Kim C. E. Leem J. (2021). A system-level mechanism of anmyungambi decoction for obesity: a network pharmacological approach. Biomolecules 11 (12), 1881. 10.3390/biom11121881 34944525 PMC8699029

[B42] Jang I. S. Yang C. S. Hwang E. H. (2007). The need for clinical practice guidelines in usage of mahuang in weight loss. J. Korean Med. Obes. Res. 7 (1), 23–29.

[B43] Keisler B. D. Hosey R. G. (2005). Ergogenic aids: an update on ephedra. Curr. Sports Med. Rep. 4 (4), 231–235. 10.1007/s11932-005-0042-4 16004835

[B44] Kim B. S. Song M. Y. Kim H. (2014). The anti-obesity effect of Ephedra sinica through modulation of gut microbiota in obese Korean women. J. Ethnopharmacol. 152 (3), 532–539. 10.1016/j.jep.2014.01.038 24556223

[B45] Kim H. Han C.-H. Lee E.-J. Song Y.-K. Shin B.-C. Kim Y.-K. (2007). A clinical practice guideline for ma-huang(ephedra sinica) prescription in obesity. J. Soc. Korean Med. Obes. Res. 7 (2), 27–37.

[B46] Kim H. J. Park J. M. Kim J. A. Ko B. P. (2008). Effect of herbal ephedra sinica and evodia rutaecarpa on body composition and resting metabolic rate: a randomized, double-blind clinical trial in Korean premenopausal women. J. Acupunct. Meridian Stud. 1 (2), 128–138. 10.1016/S2005-2901(09)60033-9 20633465

[B47] Kim J. Y. Kim D. B. Lee H. J. (2006). Regulations on health/functional foods in Korea. Toxicology 221 (1), 112–118. 10.1016/j.tox.2006.01.016 16481090

[B48] Kim K. Y. Oh J. E. (2020). Evaluation of pharmaceutical abuse and illicit drug use in South Korea by wastewater-based epidemiology. J. Hazard. Mater. 396, 122622. 10.1016/j.jhazmat.2020.122622 32388180

[B49] Kim T. Y. You S. E. Ko Y. S. (2018). Association between Sasang constitutional types with obesity factors and sleep quality. Integr. Med. Res. 7 (4), 341–350. 10.1016/j.imr.2018.06.007 30591888 PMC6303369

[B50] Kissane N. A. Pratt J. S. A. (2011). Medical and surgical treatment of obesity. Best Pract. and Res. Clin. Anaesthesiol. 25 (1), 11–25. 10.1016/j.bpa.2011.01.001 21516910

[B51] Klempel M. C. Varady K. A. (2011). Reliability of leptin, but not adiponectin, as a biomarker for diet-induced weight loss in humans. Nutr. Rev. 69 (3), 145–154. 10.1111/j.1753-4887.2011.00373.x 21348878

[B52] Lawrence J. Chen M. Xiong F. Xiao D. Zhang H. Buchholz J. N. (2011). Foetal nicotine exposure causes PKCε gene repression by promoter methylation in rat hearts. Cardiovasc Res. 89 (1), 89–97. 10.1093/cvr/cvq270 20733009 PMC3002869

[B53] Lee E. Yoon S. H. Kim H. Kim Y. D. Leem J. Park J. (2020). Ephedrae Herba in combination with herbal medicine (Zhizichi decoction and Phellodendri Cortex) for weight reduction: a case series. Integr. Med. Res. 9 (2), 100408. 10.1016/j.imr.2020.100408 32405455 PMC7210583

[B54] Leibel R. L. Rosenbaum M. Hirsch J. (1995). Changes in energy expenditure resulting from altered body weight. N. Engl. J. Med. 332 (10), 621–628. 10.1056/NEJM199503093321001 7632212

[B55] Lin Y. Hu N. Chang L. Hou M. (2012). P04.13. Population-based case-control study of Chinese herbal products containing ephedra and cardiovascular disease risk. BMC Complementary Altern. Med. 12 (1), P283. 10.1186/1472-6882-12-s1-p283

[B56] Luo T. T. Lu Y. Yan S. K. Xiao X. Rong X. L. Guo J. (2020). Network pharmacology in research of Chinese medicine formula: methodology, application and prospective. Chin. J. Integr. Med. 26 (1), 72–80. 10.1007/s11655-019-3064-0 30941682

[B57] Maglione M. Miotto K. Iguchi M. Jungvig L. Morton S. C. Shekelle P. G. (2005). Psychiatric effects of ephedra use: an analysis of food and drug administration reports of adverse events. AJP 162 (1), 189–191. 10.1176/appi.ajp.162.1.189 15625222

[B58] Ma J. yan Y. U. H. Zhao N. fang WANG Y. (2014). Clinical research of TCM Prescription“Peilian Mahuang”for simple obesity. Inf. Traditional Chin. Med. 31 (1), 46–49.

[B59] Manson J. E. Skerrett P. J. Greenland P. VanItallie T. B. (2004). The escalating pandemics of obesity and sedentary lifestyle. A call to action for clinicians. Arch. Intern Med. 164 (3), 249–258. 10.1001/archinte.164.3.249 14769621

[B60] Matsumoto M. Hirayama M. Ohtomi N. Ohno T. Nomura Y. Iida O. (2015). Influence of genetic factors on the ephedrine alkaloid composition ratio of Ephedra plants. J. Nat. Med. 69 (1), 63–67. 10.1007/s11418-014-0863-7 25115226

[B61] Maunder A. Bessell E. Lauche R. Adams J. Sainsbury A. Fuller N. R. (2020). Effectiveness of herbal medicines for weight loss: a systematic review and meta-analysis of randomized controlled trials. Diabetes, Obes. Metabolism 22 (6), 891–903. 10.1111/dom.13973 31984610

[B62] Mehendale S. R. Bauer B. A. Yuan C. S. (2004). Ephedra-containing dietary supplements in the US versus ephedra as a Chinese medicine. Am. J. Chin. Med. 32 (01), 1–10. 10.1142/S0192415X04001680 15154280

[B63] Miao S. M. Zhang Q. Bi X. B. Cui J. L. Wang M. L. (2020). A review of the phytochemistry and pharmacological activities of Ephedra herb. Chin. J. Nat. Med. 18 (5), 321–344. 10.1016/S1875-5364(20)30040-6 32451091

[B64] Mj P. Je M. Pm B. I B. Tc H. Cd M. (2021). The PRISMA 2020 statement: an updated guideline for reporting systematic reviews. BMJ Clin. Res., n71. 10.1136/bmj.n71 PMC8005924 33782057

[B65] Naik S. D. Freudenberger R. S. (2004). Ephedra-associated cardiomyopathy. Ann. Pharmacother. 38 (3), 400–403. 10.1345/aph.1D408 14742827

[B66] Ng M. Fleming T. Robinson M. Thomson B. Graetz N. Margono C. (2014). Global, regional, and national prevalence of overweight and obesity in children and adults during 1980-2013: a systematic analysis for the Global Burden of Disease Study 2013. Lancet 384 (9945), 766–781. 10.1016/S0140-6736(14)60460-8 24880830 PMC4624264

[B67] Odaguchi H. Hyuga S. Sekine M. Nakamori S. Takemoto H. Huang X. (2019). The adverse effects of ephedra herb and the safety of ephedrine alkaloids-free ephedra herb extract (EFE). Yakugaku Zasshi 139 (11), 1417–1425. 10.1248/yakushi.19-00122 31685738

[B68] Ojukwu M. Mbizo J. Leyva B. Olaku O. Zia F. (2015). Complementary and alternative medicine use among overweight and obese cancer survivors in the United States. Integr. Cancer Ther. 14 (6), 503–514. 10.1177/1534735415589347 26044767

[B69] Palamar J. (2011). How ephedrine escaped regulation in the United States: a historical review of misuse and associated policy. Health Policy 99 (1), 1–9. 10.1016/j.healthpol.2010.07.007 20685002

[B70] Park J. H. Lee M. J. Song M. Y. Bose S. Shin B. C. Kim H. J. (2012). Efficacy and safety of mixed oriental herbal medicines for treating human obesity: a systematic review of randomized clinical trials. J. Med. Food 15 (7), 589–597. 10.1089/jmf.2011.1982 22612295

[B71] Park S. J. Shon D. H. Ryu Y. H. Ko Y. (2022a). Extract of ephedra sinica stapf induces browning of mouse and human white adipocytes. Foods 11 (7), 1028. 10.3390/foods11071028 35407115 PMC8998140

[B72] Park W. Y. Song G. Boo M. Kim H. I. Park J. Y. Jung S. J. (2022b). Anmyungambi decoction ameliorates obesity through activation of non-shivering thermogenesis in Brown and white adipose tissues. Antioxidants (Basel) 12 (1), 49. 10.3390/antiox12010049 36670911 PMC9854861

[B73] Patterson A. J. Chen M. Xue Q. Xiao D. Zhang L. (2010). Chronic prenatal hypoxia induces epigenetic programming of PKC{epsilon} gene repression in rat hearts. Circ. Res. 107 (3), 365–373. 10.1161/CIRCRESAHA.110.221259 20538683 PMC2919213

[B74] Peters J. L. Sutton A. J. Jones D. R. Abrams K. R. Rushton L. (2008). Contour-enhanced meta-analysis funnel plots help distinguish publication bias from other causes of asymmetry. J. Clin. Epidemiol. 61 (10), 991–996. 10.1016/j.jclinepi.2007.11.010 18538991

[B75] Poddar K. Kolge S. Bezman L. Mullin G. E. Cheskin L. J. (2011). Nutraceutical supplements for weight loss: a systematic review. Nutr. Clin. Pract. 26 (5), 539–552. 10.1177/0884533611419859 21947637

[B76] Powell-Wiley T. M. Poirier P. Burke L. E. Després J.-P. Gorden-Larsen P. Lavie C. J. (2021). Obesity and cardiovascular disease: a scientific statement from the American heart association. Circulation 143 (21), e984–e1010. 10.1161/CIR.0000000000000973 33882682 PMC8493650

[B77] Powers M. (2001). Ephedra and its application to sport performance: another concern for the athletic trainer? J. Athl. Train. 36, 420–424. 16558668 PMC155439

[B78] Qin Z. Wu J. Xu C. Liu Z. (2019). Using meta-regression approach to explore the dose-response association between acupuncture sessions and acupuncture effects on chronic prostatitis/chronic pelvic pain syndrome. Ann. Transl. Med. 7 (6), 116. 10.21037/atm.2018.11.45 31032271 PMC6465436

[B79] Rucker D. Padwal R. Li S. K. Curioni C. Lau D. C. W. (2007). Long term pharmacotherapy for obesity and overweight: updated meta-analysis. BMJ 335 (7631), 1194–1199. 10.1136/bmj.39385.413113.25 18006966 PMC2128668

[B80] saeed W. Dhamen M. Ahmad R. Ahmad N. Naqvi A. (2019). Clinical uses and toxicity of ephedra sinica: an evidence-based comprehensive retrospective review (2004-2017). Pharmacogn. J. 11 (2), 439–444. 10.5530/pj.2019.11.68

[B81] Samenuk D. Link M. S. Homoud M. K. Contreras R. Theohardes T. C. Wang P. J. (2002). Adverse cardiovascular events temporally associated with ma huang, an herbal source of ephedrine. Mayo Clin. Proc. 77 (1), 12–16. 10.4065/77.1.12 11795249

[B82] Schwartz J. B. (2003). The influence of sex on pharmacokinetics. Clin. Pharmacokinet. 42 (2), 107–121. 10.2165/00003088-200342020-00001 12537512

[B83] Sg T. Jp H. (2002). How should meta-regression analyses be undertaken and interpreted? Statistics Med. 21 (11), 1559–1573. 10.1002/sim.1187 12111920

[B84] Shekelle P. G. Hardy M. L. Morton S. C. Maglione M. Mojica W. A. Suttorp M. J. (2003). Efficacy and safety of ephedra and ephedrine for weight loss and athletic performance: a meta-analysis. JAMA J. Am. Med. Assoc. 289 (12), 1537–1545. 10.1001/jama.289.12.1537 12672771

[B85] Shim E. B. Leem C. H. Kim J. J. Kim J. Y. (2017). Lower cellular metabolic power can be an explanation for obesity trend in Tae-Eum type: hypothesis and clinical observation. Integr. Med. Res. 6 (3), 254–259. 10.1016/j.imr.2017.06.006 28951839 PMC5605387

[B86] Song S. Tang Q. Huo H. Li H. Xing X. Luo J. (2015). Simultaneous quantification and pharmacokinetics of alkaloids in herba ephedrae-radix aconiti Lateralis extracts. J. Anal. Toxicol. 39 (1), 58–68. 10.1093/jat/bku113 25324527

[B87] Spiotta R. T. Luma G. B. (2008). Evaluating obesity and cardiovascular risk factors in children and adolescents. afp 78 (9), 1052–1058. 19007051

[B88] Sterne J. A. C. Sutton A. J. Ioannidis J. P. A. Terrin N. Jones D. R. Lau J. (2011). Recommendations for examining and interpreting funnel plot asymmetry in meta-analyses of randomised controlled trials. BMJ 343, d4002. 10.1136/bmj.d4002 21784880

[B89] Sui Y. Zhao H. L. Wong V. C. W. Brown N. Li X. L. Kwan A. K. L. (2012). A systematic review on use of Chinese medicine and acupuncture for treatment of obesity: therapeutic effect of TCM on obesity. Obes. Rev. 13 (5), 409–430. 10.1111/j.1467-789X.2011.00979.x 22292480

[B90] Sundström J. Risérus U. Byberg L. Zethelius B. Lithell H. Lind L. (2006). Clinical value of the metabolic syndrome for long term prediction of total and cardiovascular mortality: prospective, population based cohort study. BMJ 332 (7546), 878–882. 10.1136/bmj.38766.624097.1F 16510492 PMC1440609

[B91] Tang S. Ren J. Kong L. Yan G. Liu C. Han Y. (2023). Ephedrae herba: a review of its phytochemistry, pharmacology, clinical application, and alkaloid toxicity. Molecules 28 (2), 663. 10.3390/molecules28020663 36677722 PMC9863261

[B92] The Minister of Health, Labour and Welfare (2016). Japanese pharmacopoeia. 17th edn. Tokyo, Japan: Ministry of Health, Labour and Welfare.

[B93] Tziomalos K. Krassas G. E. Tzotzas T. (2009). The use of sibutramine in the management of obesity and related disorders: an update. Vasc. Health Risk Manag. 5 (1), 441–452. 10.2147/vhrm.s4027 19475780 PMC2686261

[B94] Uneda K. Kawai Y. Yamada T. Kaneko A. Saito R. Chen L. (2022). Japanese traditional Kampo medicine bofutsushosan improves body mass index in participants with obesity: a systematic review and meta-analysis. PLoS One 17 (4), e0266917. 10.1371/journal.pone.0266917 35417488 PMC9007387

[B95] WADA (2023). Prohibited list. WADA. Montreal, Canada: World Anti-Doping Agency. Available at: https://www.wada-ama.org/en/resources/world-anti-doping-program/2023-prohibited-list (Accessed September 29, 2022).

[B96] Wadden T. A. Tronieri J. S. Butryn M. L. (2020). Lifestyle modification approaches for the treatment of obesity in adults. Am. Psychol. 75 (2), 235–251. 10.1037/amp0000517 32052997 PMC7027681

[B97] Wang L. McLeod H. L. Weinshilboum R. M. (2011b). Genomics and drug response. N. Engl. J. Med. 364 (12), 1144–1153. 10.1056/NEJMra1010600 21428770 PMC3184612

[B98] Wang Y. C. McPherson K. Marsh T. Gortmaker S. L. Brown M. (2011a). Health and economic burden of the projected obesity trends in the USA and the UK. Lancet 378 (9793), 815–825. 10.1016/S0140-6736(11)60814-3 21872750

[B99] Wei P. Huo H. L. Hai M. Q. Cheng L. H. Feng X. X. Mei T. X. (2014). Pharmacokinetic comparisons of five ephedrine alkaloids following oral administration of four different Mahuang-Guizhi herb-pair aqueous extracts ratios in rats. J. Ethnopharmacol. 155 (1), 642–648. 10.1016/j.jep.2014.05.065 24929107

[B100] WHO Regional Office for Europe (2022). WHO European regional obesity report 2022.

[B101] Wooltorton E. Sibbald B. (2002). Ephedra/ephedrine: cardiovascular and CNS effects. CMAJ 166 (5), 633. 11898947 PMC99410

[B102] Yin G. Bie S. Gu H. Shu X. Zheng W. Peng K. (2020). Application of gene chip technology in the diagnostic and drug resistance detection of *Helicobacter pylori* in children. J. Gastroenterol. Hepatol. 35 (8), 1331–1339. 10.1111/jgh.14980 31930581

[B103] Yoon J. H. Park J. K. Oh S. S. Lee K. H. Kim S. K. Cho I. J. (2011). The ratio of serum leptin to adiponectin provides adjunctive information to the risk of metabolic syndrome beyond the homeostasis model assessment insulin resistance: the Korean Genomic Rural Cohort Study. Clin. Chim. Acta. 412 (23–24), 2199–2205. 10.1016/j.cca.2011.08.003 21855536

[B104] Zell-Kanter M. Quigley M. A. Leikin J. B. (2015). Reduction in ephedra poisonings after FDA ban. N. Engl. J. Med. 372 (22), 2172–2174. 10.1056/NEJMc1502505 26017843

[B105] Zhang Y. Yang M. Li Y. Liu B. Zhang L. Xiao D. (2021). Inhibition of DNA methylation in newborns reprograms ischemia-sensitive biomarkers resulting in development of a heart ischemia-sensitive phenotype late in life. Reprod. Toxicol. 105, 198–210. 10.1016/j.reprotox.2021.09.007 34536542 PMC8511209

