# Supplementary material for: Efficacy and safety of ephedra-containing oral medications: a systematic review, meta-analysis, and exploratory dose–response analysis for weight reduction
Source: Front Pharmacol. 2024 Oct 30;15:1397247. doi: 10.3389/fphar.2024.1397247 (PMC11557485; doi:10.3389/fphar.2024.1397247)
Supplement: Supplementary file 1 [file DataSheet1.pdf]

Supplementary Material

Supplementary Tables

Supplementary Table 1. Search strategy

Supplementary Table 2. Details of the intervention

Supplementary Figures

Supplementary Figure 1. Bubble plot for meta-regression on obesity related clinical outcomes

Supplementary Figure 2. Bubble plot for obesity related clinical outcomes

Supplementary Table 1. Search Strategy

1. PUBMED

#1	"Obesity"[MeSH] OR "Obesity"[Title/Abstract]<br>OR "Weight loss"[MeSH] OR "Weight loss" [Title/Abstract]<br>OR "Overweight"[MeSH] OR "Overweight"[Title/Abstract] OR "Over weight"[Title/Abstract]<br>OR "obes*"[Title/Abstract] OR "body mass index"[Title/Abstract] OR "BMI"[Title/Abstract] OR "adipos*"[Title/Abstract] OR "overeat*"[Title/Abstract] OR "over eat*"[Title/Abstract] OR "overfeed*"[Title/Abstract] OR "over feed*"[Title/Abstract] OR "overfed"[Title/Abstract] OR "over fed"[Title/Abstract] OR "weight cycling"[Title/Abstract]<br>OR "antiobesity"[Title/Abstract] OR "anti- obesity"[Title/Abstract] OR "obesitas"[Title/Abstract]<br>OR "bodyweight"[Title/Abstract] OR "body weight"[Title/Abstract] OR "body weight"[MeSH]	1,080,887<br>1,166,202*
#2	"medicine, east asian traditional"[MeSH] OR "Korean Medicine"[All] OR "Kampo*"[Title/Abstract] OR "traditional Chinese medicine"[Title/Abstract] OR "TCM"[Title/Abstract]	55,050<br>63,000*
#3	"Drugs, Chinese Herbal"[MeSH] OR "traditional Chinese herbal medicine"[Title/Abstract] OR "Chinese herb*"[Title/Abstract] OR "herbal medicine"[Title/Abstract] OR "herbal prescription"[Title/Abstract] OR "decotion"[Title/Abstract]	64,831<br>69,957*
#4	"ephedra"[MeSH] OR "ephedra sinica"[MeSH] OR "ephedra"[Title/Abstract] OR "mahuang"[Title/Abstract] OR "ma huang"[Title/Abstract]	1,228<br>1,329*
#5	#2 OR #3 OR #4	103,603<br>114,877*
#6	(randomized controlled trial [pt] OR controlled clinical trial [pt] OR randomized [tiab] OR placebo [tiab] OR drug therapy [sh] OR randomly [tiab] OR trial [tiab] OR groups [tiab]) NOT (animals [mh] NOT humans [mh])	4,797,777<br>5,167,238*
#7	#1 AND #5 AND #6	1,263<br>1,433*

2. EMBASE

#1	"Obesity"/exp OR "Obesity":ab,ti<br>OR "Weight loss"/exp OR "Weight loss":ab,ti<br>OR "Overweight"/exp OR "Overweight":ab,ti OR "Over weight":ab,ti<br>OR "obes*":ab,ti OR "body mass index":ab,ti OR "BMI":ab,ti OR "adipos*":ab,ti OR "overeat*":ab,ti OR "over eat*":ab,ti OR "overfeed*":ab,ti OR "over feed*":ab,ti OR "overfed":ab,ti OR "over fed":ab,ti OR "weight cycling":ab,ti<br>OR "antiobesity":ab,ti OR "anti- obesity":ab,ti OR "obesitas":ab,ti<br>OR "bodyweight":ab,ti OR "body weight":ab,ti OR "body weight"/exp	1,849,696<br>2,047,396*

#2	'oriental medicine'/exp OR 'chinese medicine'/exp OR 'kampo medicine'/exp OR 'korean medicine'/exp OR 'korean medicine' OR 'kampo*':ab,ti OR 'traditional Chinese medicine':ab,ti OR 'TCM':ab,ti	92,648<br>108,421*
#3	'herbaceous agent'/exp OR 'traditional Chinese herbal medicine':ab,ti OR 'Chinese herb*':ab,ti OR 'herbal medicine':ab,ti OR 'herbal prescription':ab,ti OR 'decotion':ab,ti	79,266<br>85,427*
#4	'ephedra'/exp OR 'ephedra':ab,ti OR 'mahuang'/exp OR 'mahuang':ab,ti OR 'ma huang':ab,ti	1,892<br>2,057*
#5	#2 OR #3 OR #4	152,990<br>172,828*
#6	'crossover procedure':de OR 'double-blind procedure':de OR 'randomized controlled trial':de OR 'single-blind procedure':de OR random*:de,ab,ti OR factorial*:de,ab,ti OR crossover*:de,ab,ti OR ((cross NEXT/1 over*):de,ab,ti) OR placebo*:de,ab,ti OR ((doubl* NEAR/1 blind*):de,ab,ti) OR ((singl* NEAR/1 blind*):de,ab,ti) OR assign*:de,ab,ti OR allocat*:de,ab,ti OR volunteer*:de,ab,ti	2,979,469<br>3,249,182*
#7	[animals]/lim NOT [humans]/lim	6,189,264<br>6,435,327*
#8	(#1 AND #5 AND #6) NOT #7	1,275<br>1,451*

3. Cochrane Library

#1	MeSH descriptor: [Obesity] explode all trees<br>OR MeSH descriptor: [Overweight] explode all trees<br>OR MeSH descriptor: [Weight Loss] explode all trees<br>OR MeSH descriptor: [Body Weight] explode all trees<br>OR ("obes*"OR"overweight"OR"over weight"):ab,ti,kw<br>OR ("overeat*"OR"over eat*"OR"overfeed*"OR"over feed*"OR"overfed"OR"over fed"):ab,ti,kw<br>OR ("weight cycling"OR"weight control"OR"weight reduc*"OR"weight loss"OR"body weight change*"):ab,ti,kw<br>OR ("body mass index"OR"BMI"OR"adipos*"):ab,ti,kw<br>OR ("antiobesity"OR"anti-obesity"OR"obesitas"):ab,ti,kw	104,208<br>124,469*
#2	MeSH descriptor: [Medicine, East Asian Traditional] explode all trees	1,402<br>1,726*
#3	MeSH descriptor: [Drugs, Chinese Herbal] explode all trees<br>OR MeSH descriptor: [Medicine, Chinese Traditional] explode all trees<br>OR ("Kampo*" OR " Kampo medicine*" OR "traditional Chinese medicine" OR "TCM" OR "Chinese herb*" OR "tradional Chinese Herbal medicine" OR "Chinese Herbal Drugs" OR "herbal medicine" OR "oriental medicine" OR "Chinese herb*" OR "herbal prescription" OR "decoction"):ab,ti,kw<br>OR “Korean Medicine*"	16,913<br>23,793*

#4	MeSH descriptor: [Ephedra] explode all trees<br>OR ("Ephedra*"OR"Ephedra sinica"OR"ma huang"OR"mahuang*"):ab,ti,kw	95<br>101*
#5	#2 OR #3 OR #4	16,999<br>23,837*
#6	#1 AND #5	493<br>842*

4. CINAHL

S1	TI Obesity OR AB Obesity OR MH Obesity<br>OR TI "Weight loss" OR AB "Weight loss" OR MH "Weight loss"<br>OR TI Overweight OR AB Overweight OR SU Overweight OR TI "Over weight" OR AB "Over weight"<br>OR TI obes* OR AB obes* OR TI "body mass index" OR AB "body mass index" OR TI BMI OR AB BMI OR TI adipos* OR AB adipos* OR TI overeat* OR AB overeat* OR TI "over eat*" OR AB "over eat*" OR TI overfeed* OR AB overfeed* OR AB "over feed*" OR TI "over feed*" OR TI overfed OR AB overfed OR TI "over fed" OR AB "over fed" OR TI "weight cycling" OR AB "weight cycling" OR TI antiobesity OR AB antiobesity OR TI "anti- obesity" OR AB "anti- obesity" OR TI obesitas OR AB obesitas<br>OR TI Bodyweight OR AB Bodyweight OR MH "Body Weight" OR MH "Body Weight Changes" OR TI "Body Weight" OR AB "Body Weight"	98,639<br>108,801*
S2	TI "Oriental medicine" OR AB "Oriental medicine" OR TI "East asian medicine" OR AB "East asian medicine" OR MH "Medicine, East Asian Traditional" OR TI "Chinese medicine" OR AB "Chinese medicine" OR TI "Korean medicine" OR AB "Korean medicine" OR TI Kampo* OR AB Kampo* OR TI "Traditional Chinese medicine" OR AB "Traditional Chinese medicine" OR TI TCM OR AB TCM	6,236<br>8,555*
S3	MH "Drugs, Chinese Herbal" OR TI herbaceous OR AB herbaceous OR TI "traditional chinese herbal medicine" OR AB "traditional chinese herbal medicine" OR TI "Chinese herb*" OR AB "Chinese herb*" OR TI "herbal medicine" OR AB "herbal medicine" OR TI "herbal prescription" OR AB "herbal prescription" OR TI decotion OR AB decotion	7,064<br>9,169*
S4	MH "ephedra" OR TI ephedra OR AB ephedra OR TI mahuang OR AB mahuang OR TI "ma huang" OR AB "ma huang"	154<br>193*
S5	S2 OR S3 OR S4	11,266<br>14,695*
S6	TX allocat* random* OR (MH "Quantitative Studies") OR (MH "Placebos") OR TX placebo* OR TX random* allocat* OR (MH "Random Assignment") OR TX randomi* control* trial* OR TX ((singl* n1 blind*) OR (singl* n1 mask*)) OR TX ((doubl* n1 blind*) OR (doubl* n1 mask*)) OR TX ((tripl* n1 blind*) OR (tripl* n1 mask*)) OR TX ((trebl* n1 blind*) OR (trebl* n1 mask*)) OR TX clinic* n1 trial* OR PT Clinical trial OR (MH "Clinical Trials+")	595,465<br>748,413*
	S1 AND S5 AND S6	257

		271*

5. AMED

S1	TI Obesity OR AB Obesity OR MH Obesity<br>OR TI "Weight loss" OR AB "Weight loss" OR MH "Weight loss"<br>OR TI Overweight OR AB Overweight OR SU Overweight OR TI "Over weight" OR AB "Over weight"<br>OR TI obes* OR AB obes* OR TI "body mass index" OR AB "body mass index" OR TI BMI OR AB BMI OR TI adipos* OR AB adipos* OR TI overeat* OR AB overeat* OR TI "over eat*" OR AB "over eat*" OR TI overfeed* OR AB overfeed* OR AB "over feed*" OR TI "over feed*" OR TI overfed OR AB overfed OR TI "over fed" OR AB "over fed" OR TI "weight cycling" OR AB "weight cycling" OR TI antiobesity OR AB antiobesity OR TI "anti- obesity" OR AB "anti- obesity" OR TI obesitas OR AB obesitas<br>OR TI Bodyweight OR AB Bodyweight OR MH "Body Weight" OR MH "Body Weight Changes" OR TI "Body Weight" OR AB "Body Weight"	6,023<br>8,692*
S2	TI "Oriental medicine" OR AB "Oriental medicine" OR TI "East asian medicine" OR AB "East asian medicine" OR MH "Medicine, East Asian Traditional" OR TI "Chinese medicine" OR AB "Chinese medicine" OR TI "Korean medicine" OR AB "Korean medicine" OR TI Kampo* OR AB Kampo* OR TI "Traditional Chinese medicine" OR AB "Traditional Chinese medicine" OR TI TCM OR AB TCM	3,686<br>6,106*
S3	MH "Drugs, Chinese Herbal" OR TI herbaceous OR AB herbaceous OR TI "traditional chinese herbal medicine" OR AB "traditional chinese herbal medicine" OR TI "Chinese herb*" OR AB "Chinese herb*" OR TI "herbal medicine" OR AB "herbal medicine" OR TI "herbal prescription" OR AB "herbal prescription" OR TI decotion OR AB decotion	2,056<br>3,138*
S4	MH "ephedra" OR TI ephedra OR AB ephedra OR TI mahuang OR AB mahuang OR TI "ma huang" OR AB "ma huang"	82<br>109*
S5	S2 OR S3 OR S4	5,344
S6	TX allocat* random* OR (MH "Quantitative Studies") OR (MH "Placebos") OR TX placebo* OR TX random* allocat* OR (MH "Random Assignment") OR TX randomi* control* trial* OR TX ((singl* n1 blind*) OR (singl* n1 mask*)) OR TX ((doubl* n1 blind*) OR (doubl* n1 mask*)) OR TX ((tripl* n1 blind*) OR (tripl* n1 mask*)) OR TX ((trebl* n1 blind*) OR (trebl* n1 mask*)) OR TX clinic* n1 trial* OR PT Clinical trial OR (MH "Clinical Trials+")	14,508<br>21,471*
	S1 AND S5 AND S6	34<br>43*

* Retrieved December 15, 2023

Supplementary Table 2. Details of the intervention

	Cheon, 2020	Azushima, 2015	Park, 2014[b]	Park, 2011[b]	Li, 2010(b)	Kim, 2008	XU, 2012	Park, 2013	Li, 2010(a)	Greenway, 2004	Hackman, 2006	Jian, 2004	Coffey, 2004	Boozer, 2002[a]	Boozer, 2001[a]
Ephedrae Herba	3.99	4.6%	0.6	1.2	1.2	12	2.4	3.75	1.25	0.9(8%)	0.5	14	0.75	0.15 (ephedrine)	0.72 (ephedrine)
Angelica gigantis Radix	3.99	4.6%	0.6	1.2	1.2		2.4								
Atractylodis rhizoma	3.99	7.6%	0.9996	2.01	2.01										
Glycyrrhizae Radix	2.01	7.6%	0.9996	2.01	2.01		4.0								
Scutellariae Radix		7.6%	0.9996	2.01	2.01		4.0								
Gypsum Fibrosum		7.6%	0.9996	3.0	3.0		4.0								
Rhei Rhizoma		5.7%	0.750	1.5	1.5		3.0								
Schizonepetae Herba/Spica		4.6%	0.6	1.2	1.2		2.4								
Gardeniae Fructus		4.6%	0.6	1.2	1.2		2.4								
Paeoniae Radix[c]	3.00	4.6%	0.6	1.2	1.2		2.4								
Menthae Herba		4.6%	0.6	1.2	1.2		2.4								
Ledebouriellae Radix		4.6%		1.2	1.2										
Forsythiae Fructus		4.6%	0.6	1.2	1.2		2.4								
Zingiberis Rhizoma		1.1%	0.2004	1.2	1.2		0.8								
Platycodi Radix		7.6%	0.9996	2.01	2.01		4	3.75	1.25						
Coicis Semen	9.99							11.25	3.75						
Cinnamomi cortex	3.00														
Cnidii Rhizoma		4.6%		1.2	1.2		2.4								
Chuanxiong Rhizoma			0.6												

Kadinum		11.4%													
Natrii Sulfas		2.7%	0.3756	1.5	1.5		1.5								
Saposhinikoviae Radix			0.6				2.4								
Castaneae Semen								11.25	3.75						
Talcum Crystallinnumm			1.5	5.01	5.01		6								
Corn starch						1									
Sodium sulfate							1.5								
Raphani Semen								7.5	2.50						
Schisandrae Fructus								3.75	1.25						
Liriopis tuber								3.75	1.25						
Acori Tatainowii Rhizoma								3.75							
Acori Graminei Rhizoma									1.25						
Coptidis Rhizoma[d]												40			
Eupatorii Herba												50			

- The unit of daily dose is described with g. Every material in this table are described with biopharmaceutical name

- Hioki 2014 are excluded because there’s no information about daily dose.

- Other materials in dietary supplement are not included in this table (boozer 2002, boozer 2001)

[a] Boozer 2002 and Boozer 2001 are described with the dosage of ephedrine because of no information about dosage of ephedra.

[b] The composition of intervention of Park 2014 and Park 2011 is followed by Bangpoongtongsungsan Ex. Granule from Hanpoong Co.,Ltd.

https://nedrug.mfds.go.kr/pbp/CCBBB01/getItemDetail?itemSeq=197900572.

[c] Paeoniae Radix includes Paeoniae Radix alba.

[d] Coptis chinensis Franch is classified with Coptidis Rhizoma.

Supplementary Figure 1. Bubble plot for meta-regression on obesity related clinical outcomes

A. BMI Change

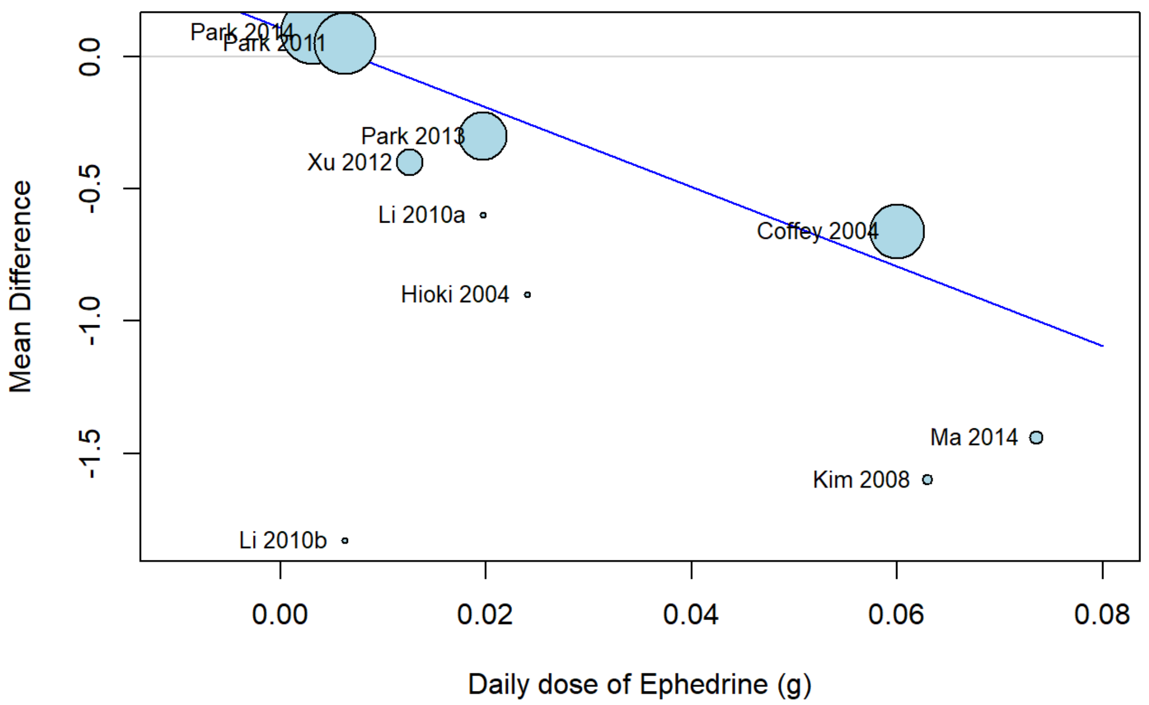

This figure above is a bubble plot representing the meta-regression analysis, which shows that the daily dose of Ephedrine can explain the heterogeneity of meta-analysis result concerning BMI change (coefficient=-15.0705, P<.0001).

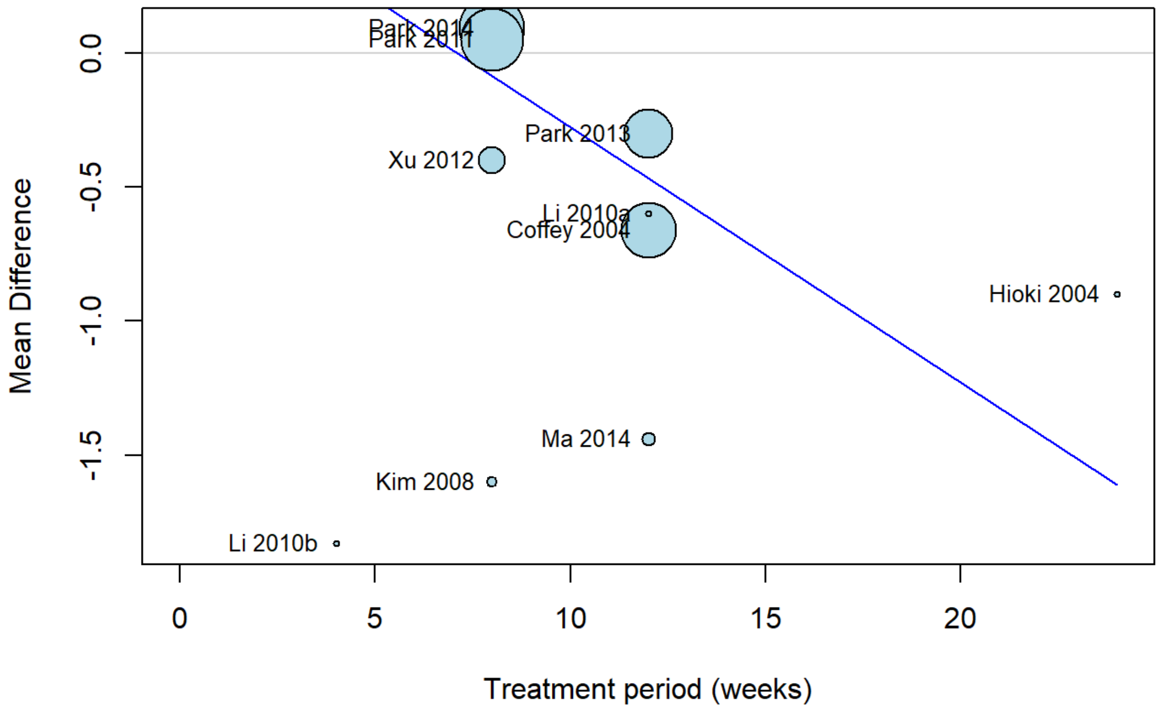

This figure above is a bubble plot representing the meta-regression analysis, which shows that treatment period can explain the heterogeneity of meta-analysis result concerning BMI change (coefficient=-0.0953, P=0.0347).

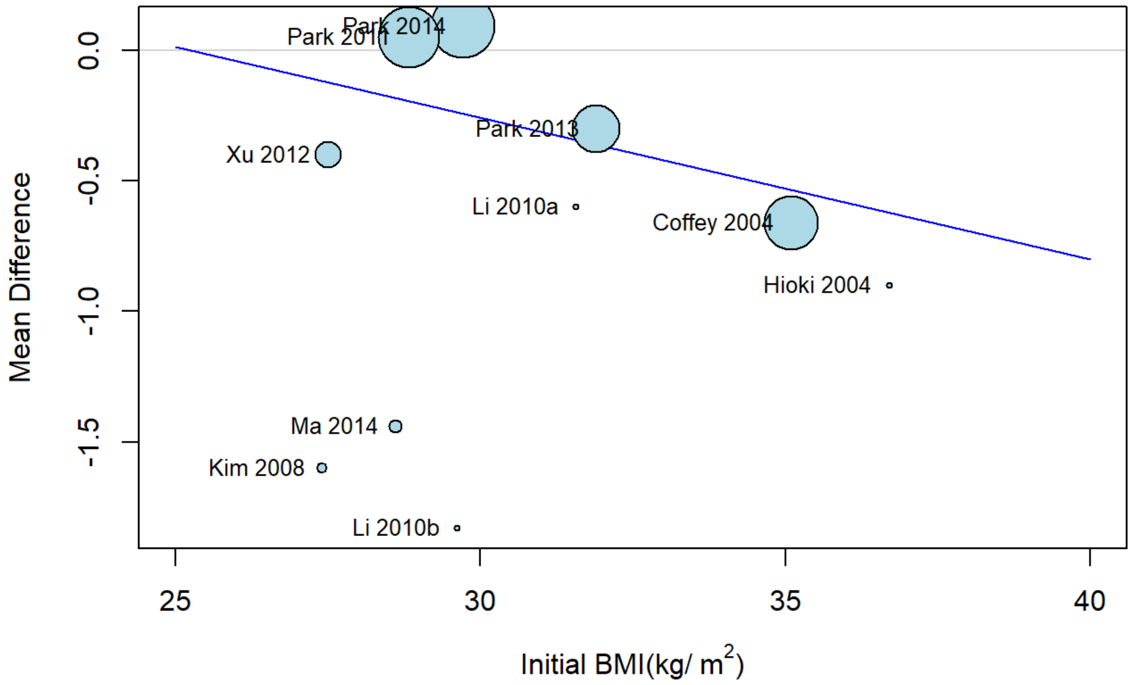

This figure above is a bubble plot representing the meta-regression analysis, which shows that initial BMI doesn't explain the heterogeneity of meta-analysis result concerning BMI change (coefficient=-0.0541, P=0.3005).

B. BW change

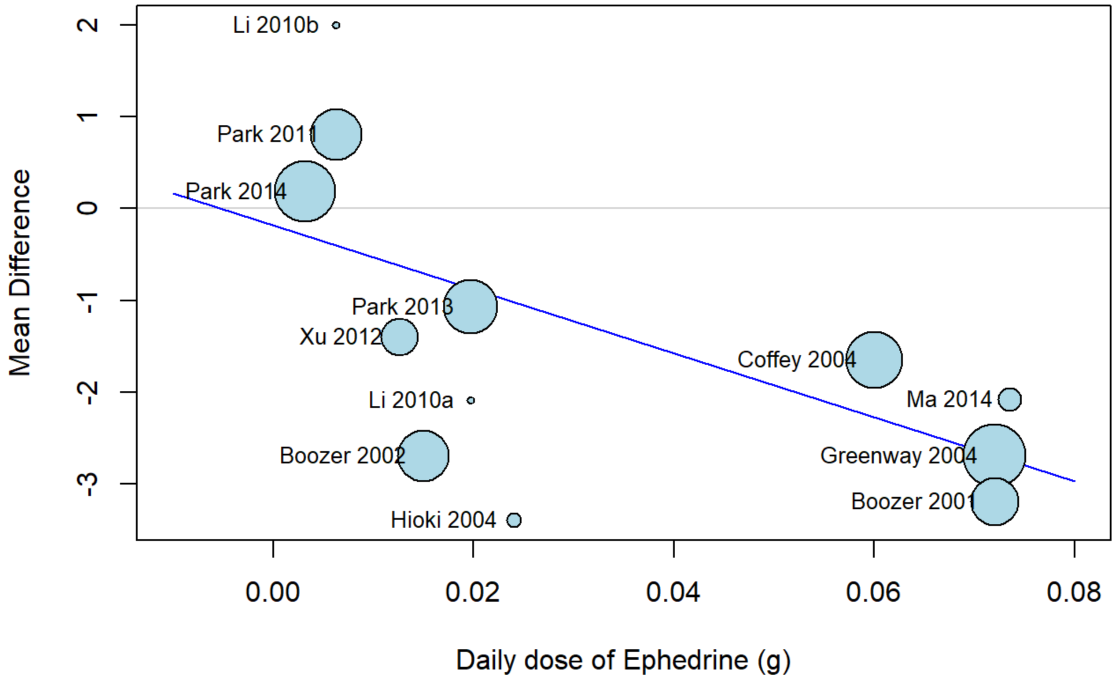

This figure above is a bubble plot representing the meta-regression analysis, which shows that daily dose of Ephedrine can explain the heterogeneity of meta-analysis result concerning BW change (coefficient=-34.9725, P=0.0002).

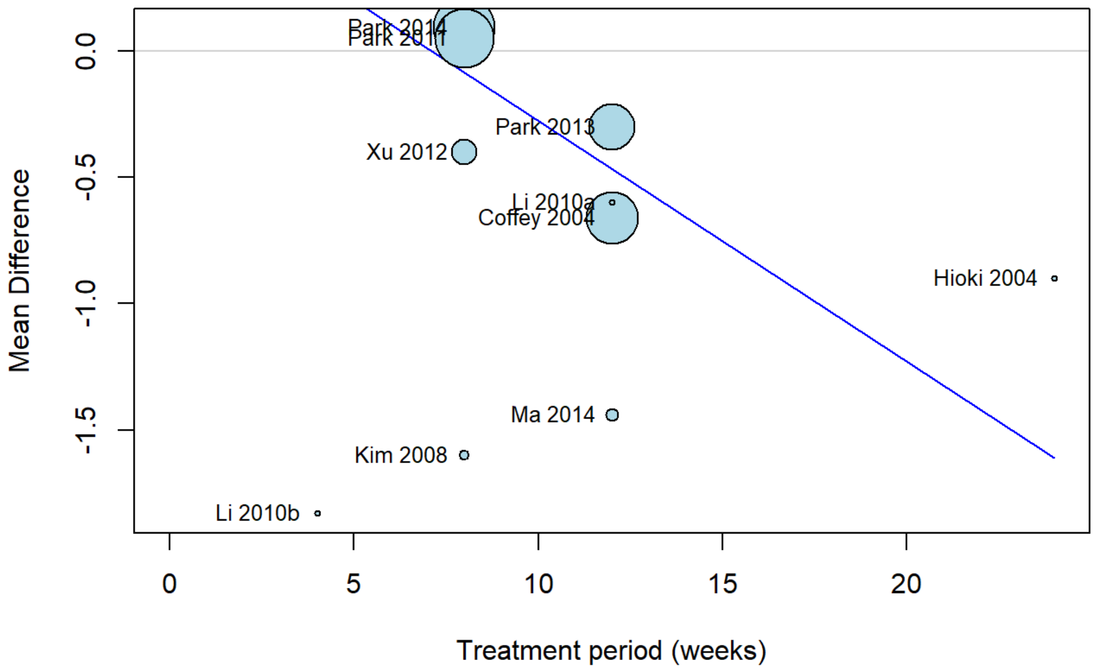

This figure above is a bubble plot representing the meta-regression analysis, which shows that treatment period doesn't explain the heterogeneity of meta-analysis result concerning BW change (coefficient=-0.1415, P=0.1242).

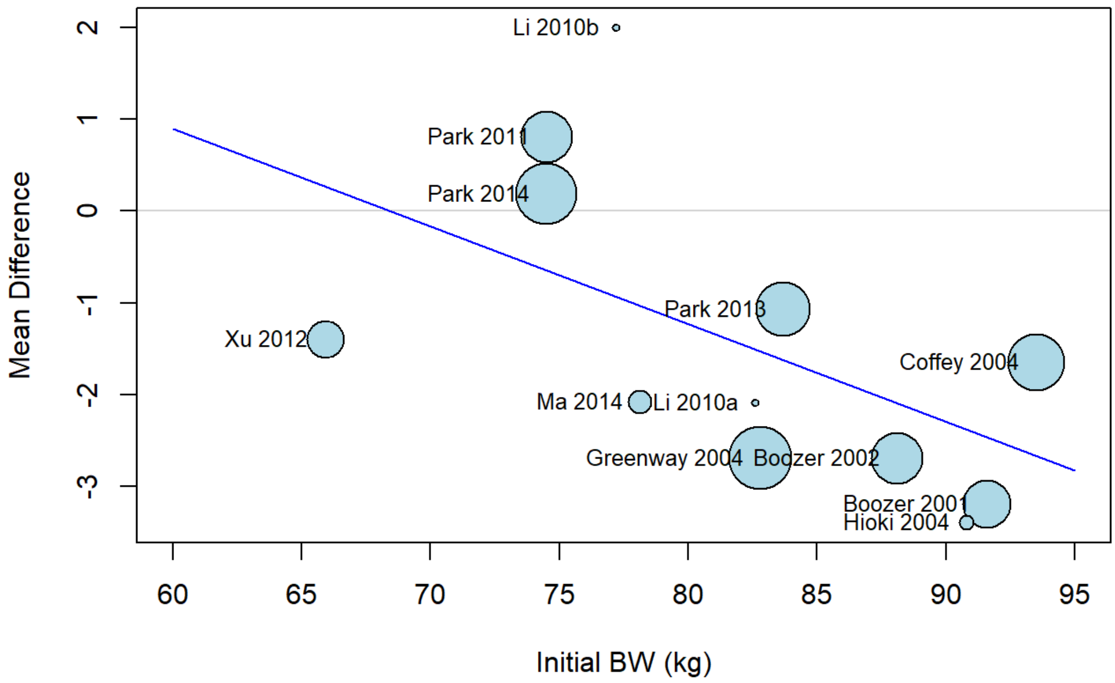

This figure above is a bubble plot representing the meta-regression analysis, which shows that initial BW doesn't explain the heterogeneity of meta-analysis result concerning BW change (coefficient=-0.1065, P=0.0639).

C. WC change

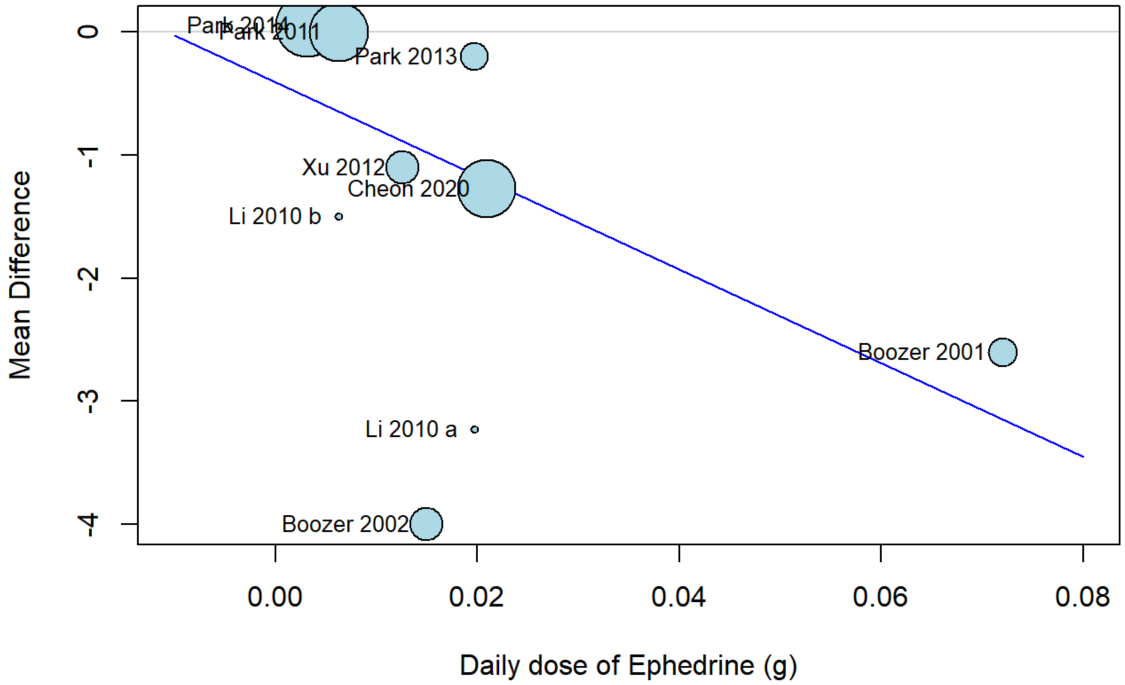

This figure above is a bubble plot representing the meta-regression analysis, which shows that daily dose of Ephedrine doesn't explain the heterogeneity of meta-analysis result concerning WC change (coefficient=-38.0242, P=0.0960).

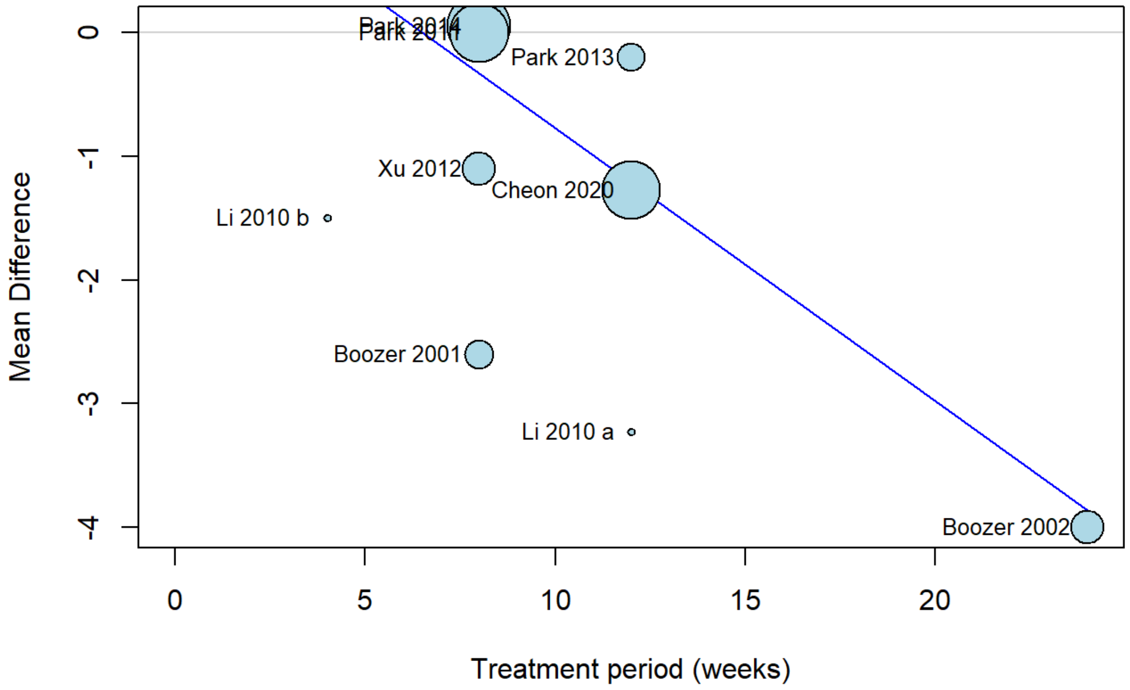

This figure above is a bubble plot representing the meta-regression analysis, which shows that treatment period can explain the heterogeneity of meta-analysis result concerning WC change (coefficient=-0.2211, P=0.0017).

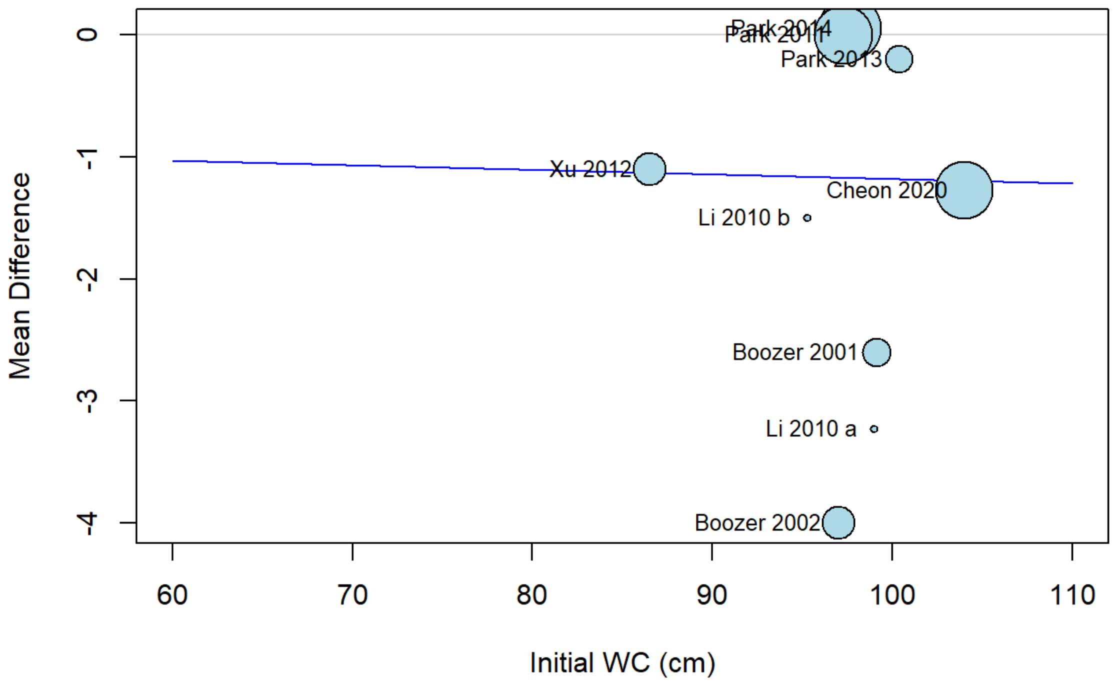

This figure above is a bubble plot representing the meta-regression analysis, which shows that initial WC does not explain the heterogeneity of meta-analysis result concerning WC change (coefficient=-0.0038, P=0.9724).

Supplementary Figure 2. Bubble plot for obesity related clinical outcomes

A. BMI Change

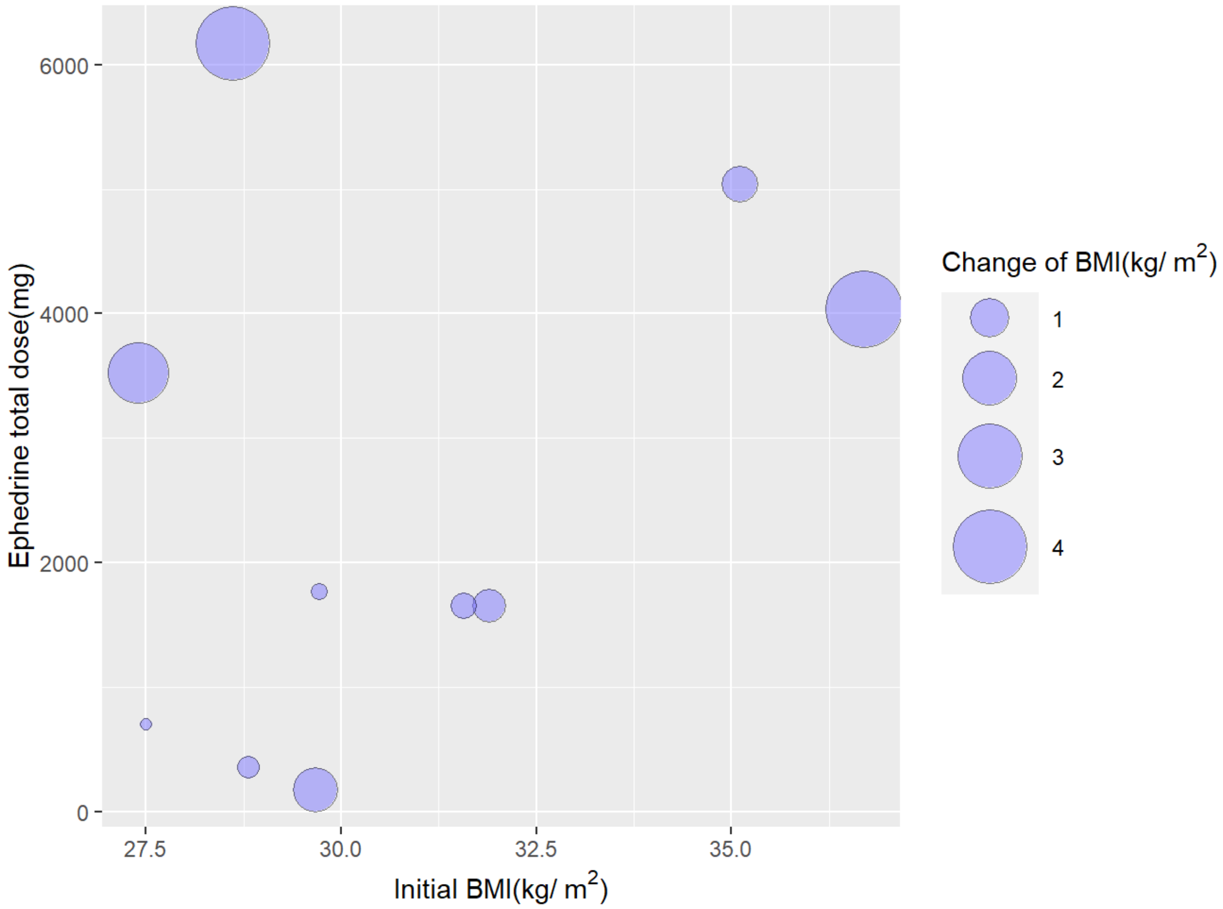

B. BW change

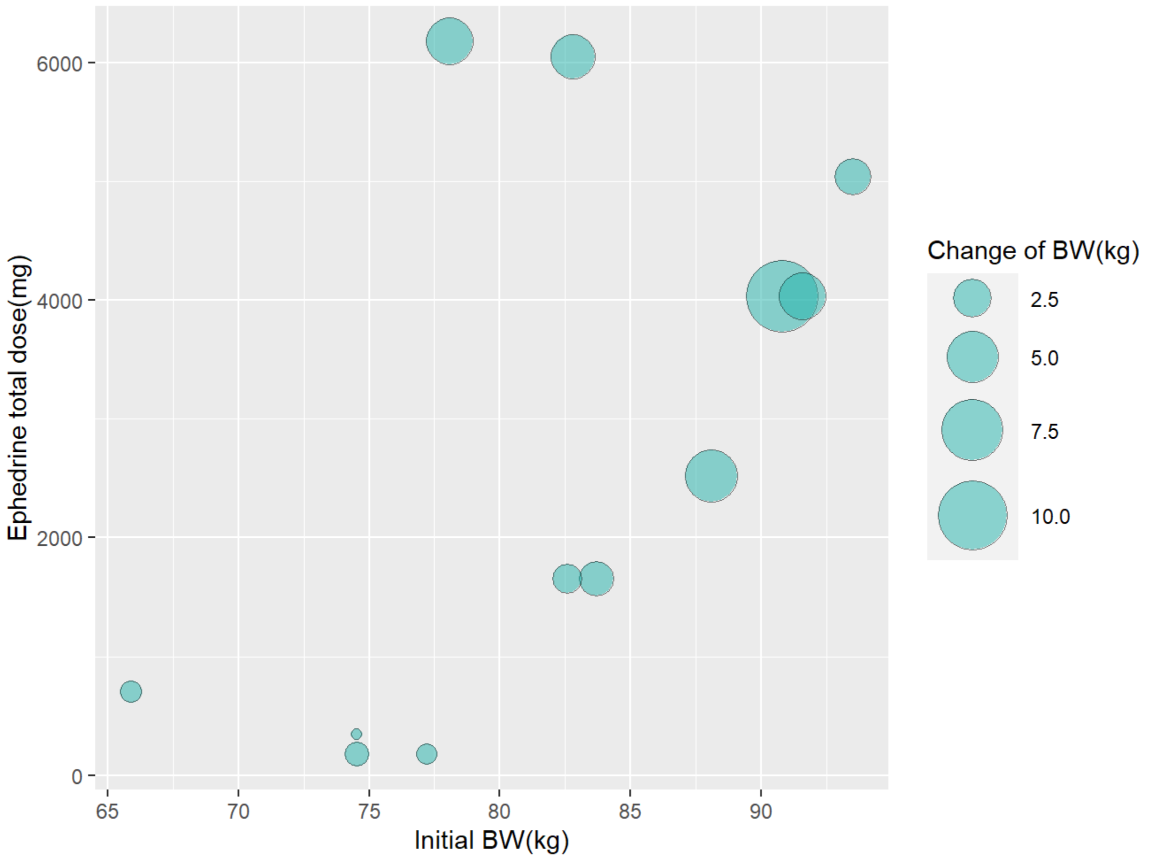

Ephedrine total dose(mg)
6000
4000
2000
0
65
70
75
80
85
90
Initial BW(kg)
Change of BW(kg)
2.5
5.0
7.5
10.0

C. WC change

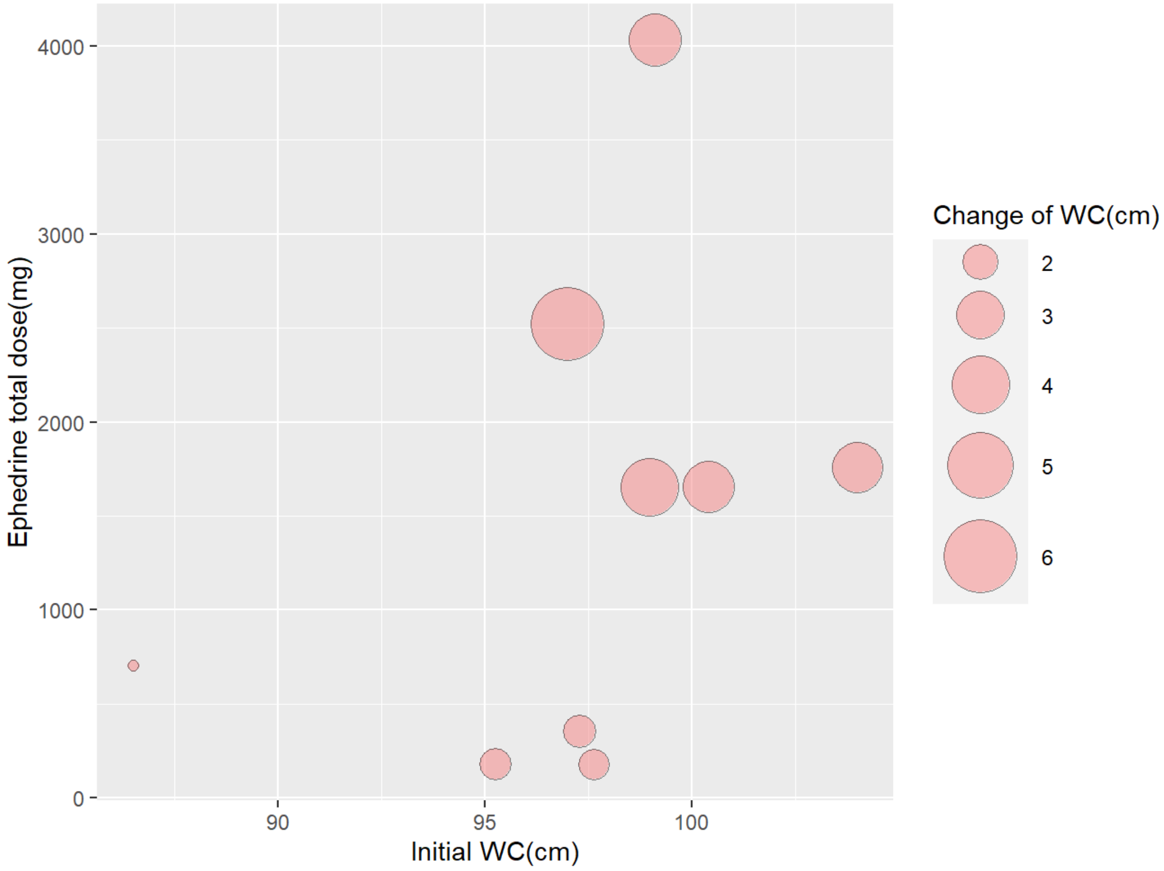

Ephedrine total dose(mg)
4000
3000
2000
1000
0
90
95
100
Initial WC(cm)
Change of WC(cm)
2
3
4
5
6
